# CD8^+^ T cells cross-restricted by HLA-B*57 and HLA-E*01 recognize HIV Gag with different functional profiles

**DOI:** 10.1172/jci.insight.189909

**Published:** 2025-12-18

**Authors:** Kevin J. Maroney, Michael A. Rose, Allisa K. Oman, Abha Chopra, Hua-Shiuan Hsieh, Zerufael Derza, Rachel Waterworth, Mark A. Brockman, Spyros A. Kalams, Anju Bansal, Paul A. Goepfert

**Affiliations:** 1 Department of Medicine, Division of Infectious Diseases, School of Medicine, University of Alabama at Birmingham, Birmingham, Alabama, USA.; 2 Centre for Molecular Medicine and Innovative Therapeutics, Health Futures Institute, Murdoch University, Perth, Western Australia, Australia.; 3 Faculty of Health Sciences, Simon Fraser University, Burnaby, British Columbia, Canada.; 4 Department of Medicine, Division of Infectious Diseases, Vanderbilt University Medical Center, Nashville, Tennessee, USA.

**Keywords:** AIDS/HIV, Infectious disease, Adaptive immunity, Antigen, T cell receptor

## Abstract

Few HIV-specific epitopes restricted by non-classical HLA-E have been described, and even less is known about the functional profile of responding CD8^+^ T cells (CD8s). This study evaluates the functional characteristics of CD8s targeting the Gag epitope KF11 (KAFSPEVIPMF) restricted by either HLA-E (E-CD8s) or HLA-B57 (B57-CD8s). CD8s from 8 people with HIV (PWH) were cocultured with KF11 peptide presented by cell lines expressing HLA-B*57:01, HLA-E*01:01, or HLA-E*01:03. CD8 responses were analyzed using single-cell RNA and TCR sequencing. Supernatants were also assessed for soluble protein profiling. HLA-I multimers were developed to identify CD8s restricted by HLA-B57 and/or HLA-E ex vivo. B57-CD8s secreted higher levels of cytotoxic cytokines such as IFN-γ, whereas E-CD8s produced more chemotactic cytokines, including RANTES, CXCL10 (IP-10), and IL-27, findings that were corroborated through single-cell RNA sequencing. TCR clonotypes stimulated by KF11 were cross-restricted by HLA-B*57 and HLA-E*01:03 as demonstrated by in vitro T cell reporter assays and ex vivo multimer screening. Ex vivo CD8s were singly restricted by HLA-B57 and HLA-E, with dual restriction only observed in PWH with lower viral load. These findings demonstrate that certain HIV-specific CD8s in PWH exhibit dual restriction by HLA-B*57 and HLA-E*01:03, leading to functionally distinct immune responses depending on the restricting allele(s).

## Abstract 2

This work suggests that bi restriction of the same TCR by both HLA-Ia and -E is important for the host response to HIV infection.

## Graphical abstract

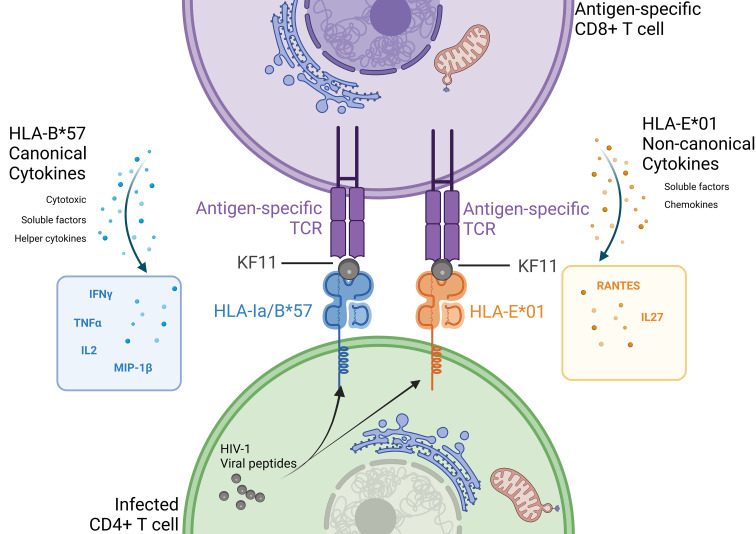

## Introduction

Seminal studies demonstrating that vaccine-induced, Mamu-E–restricted CD8^+^ T cells (CD8s) can prevent the establishment of SIV infection have generated interest in the potential of HLA-E–restricted responses in HIV research (1, 2). CD8s restricted by non-classical HLA molecules may be particularly advantageous for vaccine design, as only two HLA-E alleles (E*01:01 and E*01:03) need to be considered, each occurring at approximately 50% frequency in human populations (1–4).

We previously reported that the immunodominant HIV-1 epitope KF11 (KAFSPEVIPMF, Gag [162–172]), classically presented by the controller-associated, classical HLA-B*57 allele, can also elicit CD8^+^ T cell responses restricted by HLA-E (5). This finding is supported by a separate report of de novo HLA-E–restricted CD8^+^ T cell responses to RL9 (RMYSPTSIL, Gag [275–283]) (6), confirming that HLA-E can present HIV-1–derived epitopes to human CD8s. Nonetheless, HLA-E–restricted CD8^+^ T cell responses remain understudied in the context of HIV infection.

HLA-E–restricted CD8^+^ T cell responses may also contribute to control of HIV infection. However, identifying HIV-derived HLA-E–restricted epitopes has been challenging, largely because of their extremely low affinity for HLA-E (6–8), especially in contrast to immunogenic epitopes derived from *Mycobacterium tuberculosis* (Mtb) or human cytomegalovirus (HCMV) (9).

To better understand the role that HLA-E– and HLA-B57–restricted CD8s (E-CD8s and B57-CD8s) play in chronic HIV-1 infection, we used targeted single-cell RNA and T cell receptor sequencing (scRNA-seq and scTCR-seq) complemented by a high-parameter Luminex assay. This approach helped identify shared and unique features of KF11-specific CD8s restricted by these alleles. We found that KF11-specific CD8s can be cross-restricted by both HLA-B*57 and HLA-E (E*01:01 and/or E*01:03). Furthermore, scTCR-seq and in vitro TCR reporter assays (10) indicated that certain TCR clonotypes displayed cross-restriction for KF11 presented by both HLAs. Despite this cross-restriction, we observed functionally distinct secretory protein and RNA expression profiles between CD8s stimulated by KF11 in the context of HLA-B57 or HLA-E. In order to determine whether these phenomena can be observed ex vivo, we also developed HLA-E/B57 multimer technology, and were able to confirm their functional distinction, as well as dual restriction in the controllers.

## Results

### Study design.

We analyzed CD8^+^ T cell responses of 8 people with HIV (PWH), categorized as follows: one antiretroviral therapy–naive (ART-naive) elite controller (EC), four ART-naive controllers (C), and 2 ART-naive non-controllers (NC), with plasma viral loads <200, <2,000, or >2,000, respectively, as well as one ART-suppressed individual with viral suppression (ART+ NC). All patients expressed HLA-B*57 and displayed KF11-specific responses by IFN-γ ELISPOT assay. CD8s from each participant were cocultured with single-HLA-expressing target cells (either HLA-B*57, -E*01:01, or -E*01:03) loaded with KF11 peptide (Supplemental Figure 1; supplemental material available online with this article; https://doi.org/10.1172/jci.insight.189909DS1). Antigen-specific CD8s (identified as dual CD69^+^CD137^+^) were then sorted by flow cytometry and analyzed via SMART-seq2–based scRNA-seq and scTCR-seq. Supernatants were also assessed using a high-parameter Luminex assay. For a subset (one each of C, ART+ NC, and NC), we performed 10x Genomics–based scRNA-seq, TCR-seq, and CITE-seq analysis. We also confirmed HLA-E and/or -B57 restriction for CD8s ex vivo (not in 18-hour in vitro assay) by multimer.

### Cytokine profiles of B57- and E01-CD8s are functionally distinct.

**Figure 1:**
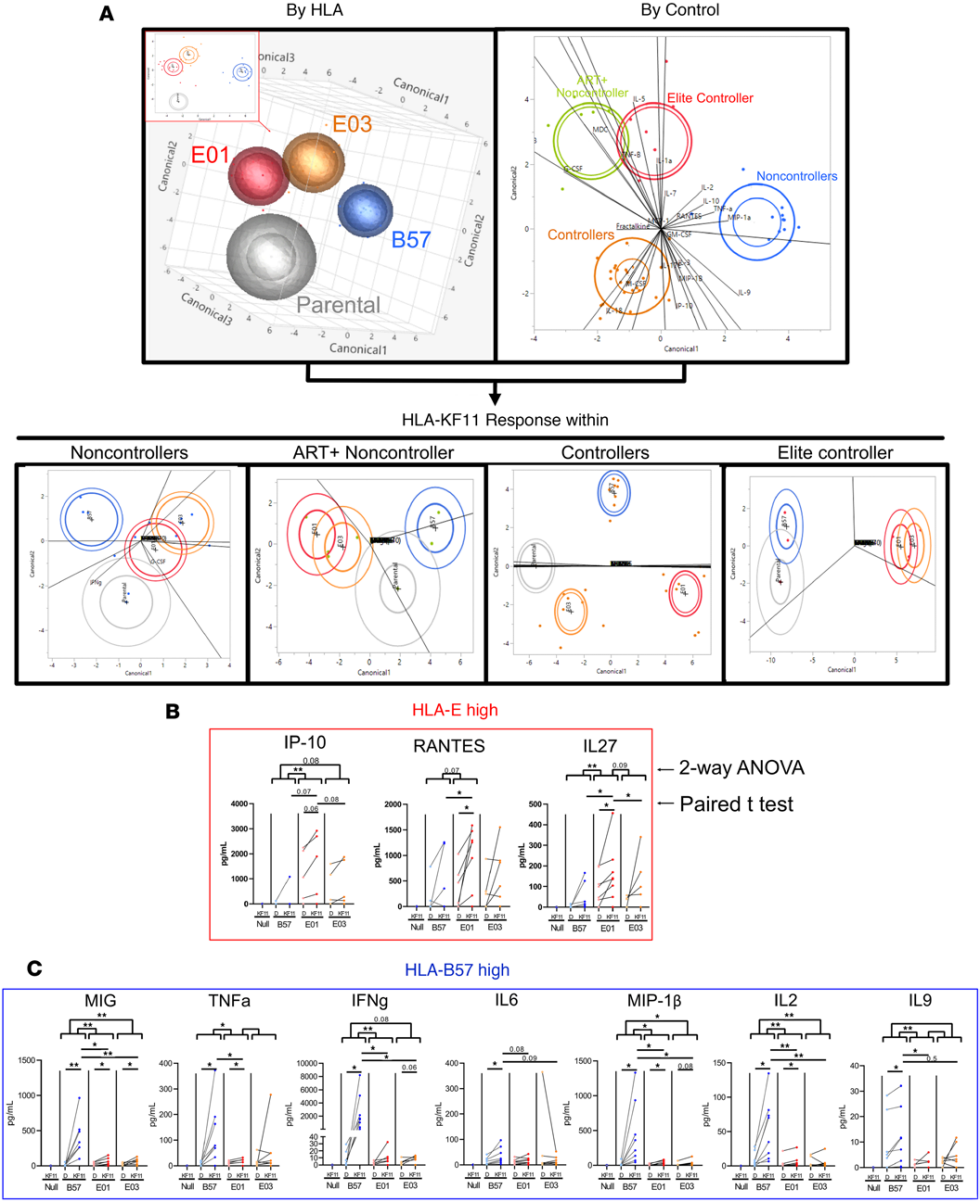
Luminex data show distinct functionality of E- versus B57-CD8s. (**A**) Determinant analysis for clustering by HLA, by control, or by HLA within control subsets of all samples using all cytokine concentrations. (**B** and **C**) Individual cytokines that significantly increase from DMSO (“D” in graph) to KF11 conditions and/or show a significant difference between HLA conditions by 2-way ANOVA from **A** within each HLA are represented as bar graphs and grouped by cytokines that are HLA-E high (**B**) or HLA-B57 high (**C**). Both the 2-way ANOVA significance indicated from **A** and paired *t* test values in **B** and **C** are indicated as follows: *P < 0.05, **P < 0.01.

Using a 38-plex human cytokine/chemokine panel, we assessed cytokine secretion following KF11 stimulation with HLA-B*57 or HLA-E*01:01/03 (Supplemental Figure 2). Secreted proteomes formed functionally distinct spatial clusters by HLA on the top 3 component groupings of cytokines (Figure 1A). Additionally, responses also clustered distinctly by HIV control status, with ART+ NC clustering more closely with the EC than other ART-naive NCs (Figure 1A). The greatest HLA-restricted functional distinctions were observed in controllers and the EC, indicating increasing divergence in cytokine profiles with lower viral loads (Figure 1A).

Of the cytokines examined, CXCL10 (IP-10), CCL5 (RANTES), and IL-27 were significantly increased in E-CD8s (Figure 1B), while CXCL9 (MIG), TNF-α, IFN-γ, IL-6, CCL4 (MIP-1β), IL-2, and IL-9 were significantly elevated in B57-CD8s, by 2-way ANOVA (Figure 1C). Antigen specificity was confirmed by paired *t* test between DMSO and KF11 conditions. Two of these three E-CD8 cytokines and four of seven B57-CD8 cytokines were then confirmed to be transcribed in CD8 subsets upon 10x Genomics–based single-cell assays (Supplemental Figure 3).

### TRAV5*01 metaclonotype cluster cross-restricted in controllers and ART+ non-controller.

**Figure 2:**
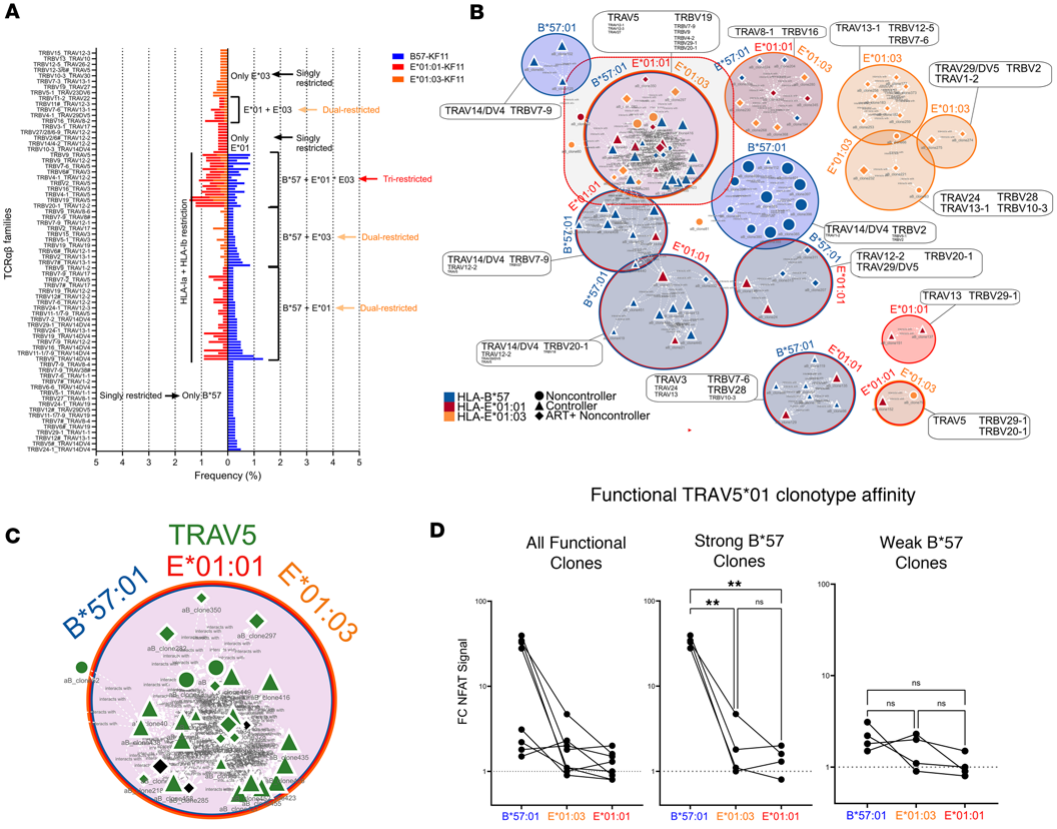
Single-cell-sorted activated CD8 clonotypes are cross-restricted. (**A**) Pyramid plot representing total proportion of all TCR Vαβ family pairs observed in response to HLA-B*57:01–restricted (blue), HLA-E*01:01–restricted (red), and HLA-E*01:03–restricted (orange) KF11. Those pairs that were observed in 2 or 3 HLA restriction states are indicated as dual- or tri-restricted with an arrow and bracket. (**B**) Clusters of similar HLA-B*57:01–, -E*01:01–, and -E*01:03–restricted clonotypes are colored in blue, red, and orange, respectively. Clonotypes identified in non-controllers are represented as circles, while those in controllers and the ART-suppressed non-controller are represented as triangles and diamonds, respectively. Each cluster is labeled with the HLA-restricted state most common in the given cluster and colored accordingly. Additionally, every TRAV and TRBV family included in each is indicated for each cluster with font size scaled by prevalence. (**C**) The tri-restricted metaclonotype cluster containing the highest proportion of highly similar clonotypes from all 3 HLA restriction states is colored in green for all clonotypes that have TRAV5, while those that have a different α family are colored in black. (**D**) Line graph of fold change in functional affinity for TRAV5-containing complementarity-determining region 3 (CDR3) αβ pairs cloned into a Jurkat reporter cell line and co-incubated with HLA-B*57:01– or HLA-E*01:01–transduced HLA-null line pulsed with KF11. Fold change is for indicated HLA over HLA-A*02 cell line co-incubation pulsed with KF11. **P < 0.01 by Tukey’s multiple-comparison test.

We sorted KF11-specific CD8s to perform scRNA/TCR-seq (Supplemental Figure 4). Single KF11-specific T cells activated in their HLA-restricted condition over null line coculture were sorted into 96-well plates by FACS and sequenced. Based on paired TCR α/β variable gene sequences, most responses across all samples appeared to be cross-restricted (i.e., the same gene pairs were isolated from cells stimulated by B57 and E) (Figure 2A). This phenomenon was also observed at an individual PWH level (Supplemental Figure 5).

To account for the inherent heterogeneity of TCR variable chain sequences between different individuals (even those specific for the same antigen), we applied a sequence similarity–based approach (implemented in TCRdist3) (11) to cluster highly related clones into metaclonotypes (Figure 2B). We observed clusters of TCRs singly restricted by HLA-E*01:01, HLA-E*01:03, or HLA-B*57:01, dually restricted by HLA-E*01:01 and -B*57:01, and a large cluster of tri-restricted clonotypes enriched for controller and ART+ NC TCRs. The tri-restricted cluster specifically was enriched for TRAV5 and TRBV19 (Figure 2C). ART-naive NC clones were primarily identified as being mono-B*57-restricted (Figure 2B). The cross-restriction of TRAV5-containing clones was confirmed using an in vitro luciferase reporter cell assay, comparing functional avidity to HLA-restricted KF11 over KF11-loaded HLA-A*02 negative controls (Figure 2D). TCR signaling intensity was reduced when KF11-pulsed target cells expressed either E01:01 or E01:03, compared with B*57, suggesting lower peptide affinity of KF11 for HLA-E as compared with canonical HLA-B*57; however, even these responses were significantly elevated over HLA-A*02:01 KF11-pulsed controls for the same clones.

### Cross-restricted TCR clonotypes express transcriptional signatures consistent with higher metabolic activity.

**Figure 3:**
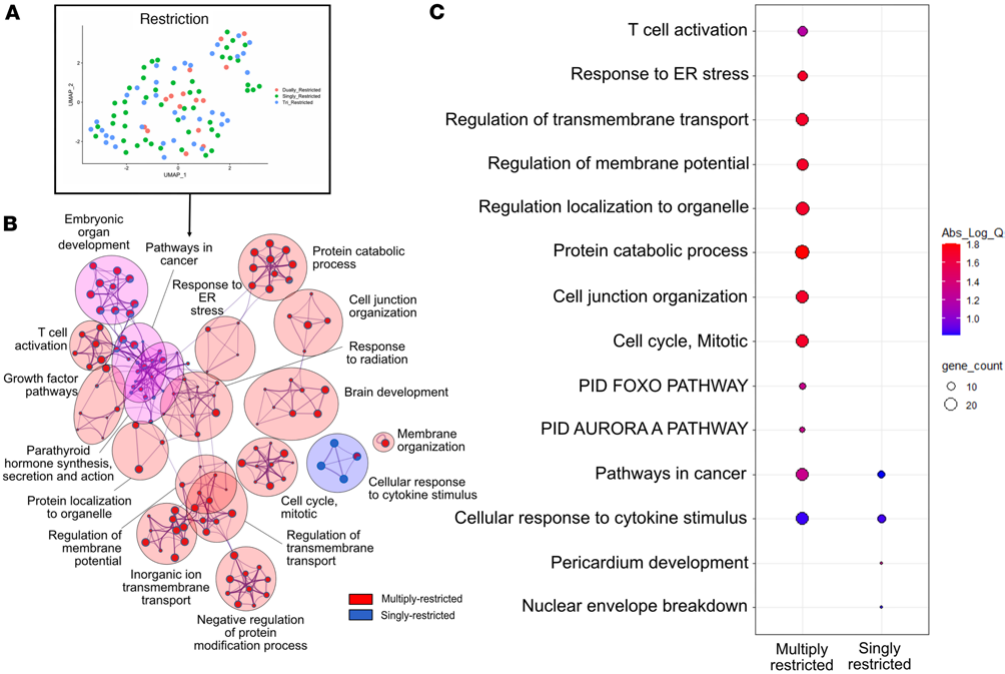
Multiply-restricted CD8s exhibit signatures of higher metabolic activity. (**A**) Principal component analysis of highest-proportion CD8s in single-, dual-, and tri-restricted metaclonotype clusters of **B** and **C**, colored by membership. (**B**) Gene Ontology (GO) bubble map of CD8s from **A**, with relative percentage of enriched genes contributing to each GO node represented as a pie chart. Colors map to gene ontology nodes representing the enriched set of genes higher in either “Multiply-restricted” or “Singly-restricted” metaclonotype cluster CD8s as indicated in figure key. Analysis was performed using Metascape (https://metascape.org). (**C**) Relative enrichment for each enriched set of multiply- versus singly-restricted comparison for the indicated GO terms as a bubble map. Size of circles correlates with confidence and significance of the enriched set as measured by absolute log(*Q*) value to the GO term. This is based on both the number and types of genes included in the gene list of the indicated GO term; the number is indicated by color as shown in the inset legend.

To determine transcriptional features associated with cross-restriction, we stratified clonotypes according to their presence in tri-restricted, dually restricted, or singly restricted clusters and then performed model-based analysis of single-cell transcriptomics (MAST) (12) on the linked scRNA-seq data for each clonotype’s CD8s (Figure 3). While the transcriptional profiles of T cells in each type of cluster did not cluster separately by the most variable genes (Figure 3A), when compared with singly restricted clones directly, the multiply-restricted clones displayed upregulation of metabolism-associated genes associated with protein catabolic activity, mitosis, and T cell activation pathways (Figure 3B). In fact, most transcriptional signatures were higher in CD8s that were multiply restricted (Figure 3C).

### Single-cell transcriptional expression clusters distinctly depending on HLA restriction.

To obtain an in-depth single-cell-level resolution of molecular signatures delineating E-CD8s from B57-CD8s, we subjected 3 PWH (one C, one ART+ NC, and one ART-naive NC; selected based on sample availability) to 10x Genomics single-cell TCR- and CITE-seq. We adopted a broader sorting strategy with multiplexed conditions to assess memory T cell subsets and markers that may differentiate E-restricted from B57-restricted CD8s. In short, we performed 10x scRNA-seq, TCR-seq, and CITE-seq on enriched populations of activated and non-activated CD8s from all conditions (Supplemental Figure 1). Demultiplexing, pre-processing, and quality control of the data were carried out using Seurat as described in Methods (Supplemental Figure 6).

**Figure 4:**
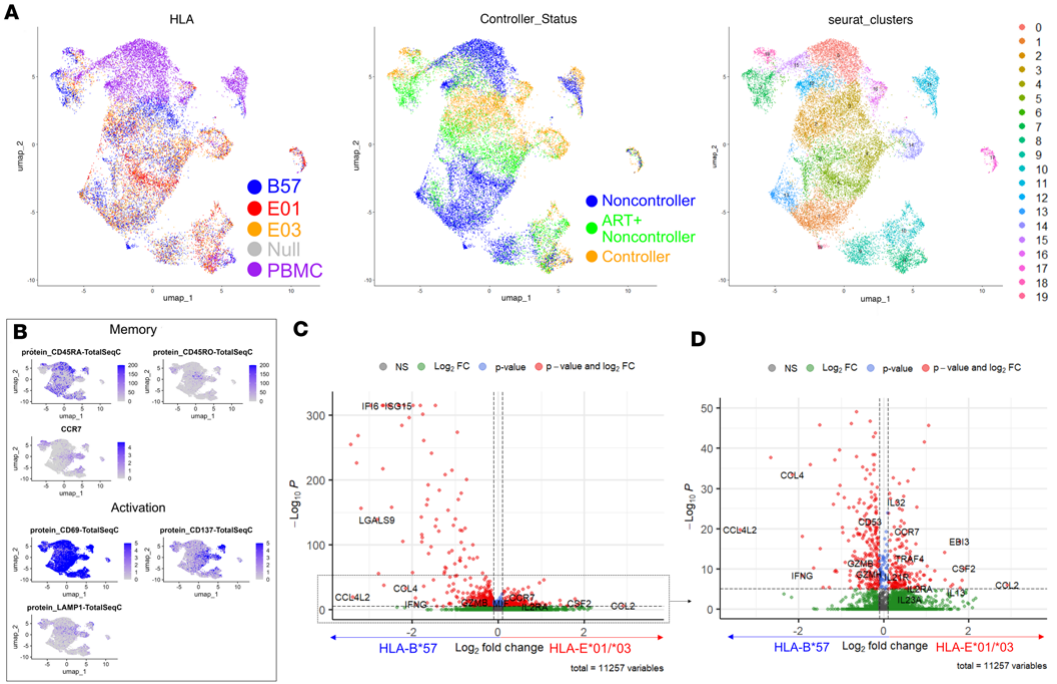
CD8s cluster mainly by control and HLA restriction status. 10x Genomics–based transcriptomic and proteomic analysis of activated and unactivated CD8s sorted from 41A3.CD4.[HLA] line coculture or PBMCs of *n* = 1 non-controller, controller, and ART+ non-controller, which were stimulated or unstimulated with KF11 peptide. (**A**) UMAP DimPlots for metadata distribution of HLA (left), controller status (middle), and Seurat cluster assignment (right) from all sorted CD8s colored according to the inset legends. (**B**) Feature plots show CITE-seq enrichment for memory or activation markers within dataset as indicated, used for later metadata assignment by quantile expression. (**C**) Default frame volcano plot (maximum –log[*P* value] of 300) for differentially expressed genes identified by MAST between B57-CD8s and all E01/E03-CD8s. Significant markers above cutoff *P* value and fold change are colored in red. (**D**) “Zoomed-in” view of **C** with maximum *P* value of 50.

CD8s primarily clustered by HIV controller status and HLA restriction in Seurat uniform manifold approximation and projection (UMAP) representation (Figure 4A). We also observed through CITE-seq that CD45RA and CD45RO, the activation markers CD69, CD137, and LAMP1, and RNA expression of the chemokine CCR7 were present within distinct clusters of cells, especially CD45RA versus CD45RO, validating their fidelity (Figure 4B). The most variably expressed genes were also predominantly immune related (CCL4, XCL1, IL-2, XCL2, IL-13, etc.) and served to differentiate distinct clusters (Supplemental Figure 7, A and B). Pairwise MAST analysis between B57-CD8s and grouped E-CD8s (E*01:01/E*01:03) confirmed upregulation of previously identified secreted HLA-restricted cytokines, including EBI3 (a subunit of IL-27) as well as CCL4 and IFN-γ, upregulated in E-CD8s or B57-CD8s, respectively (Figure 4, C and D). Two of three E-CD8–associated and over half of B57-CD8–associated cytokines were also upregulated in corresponding CD8 subsets (Supplemental Figure 3).

### Antigen-specific TCRs identified through VCR-Seek bioinformatic pipeline are all cross-restricted.

**Figure 5:**
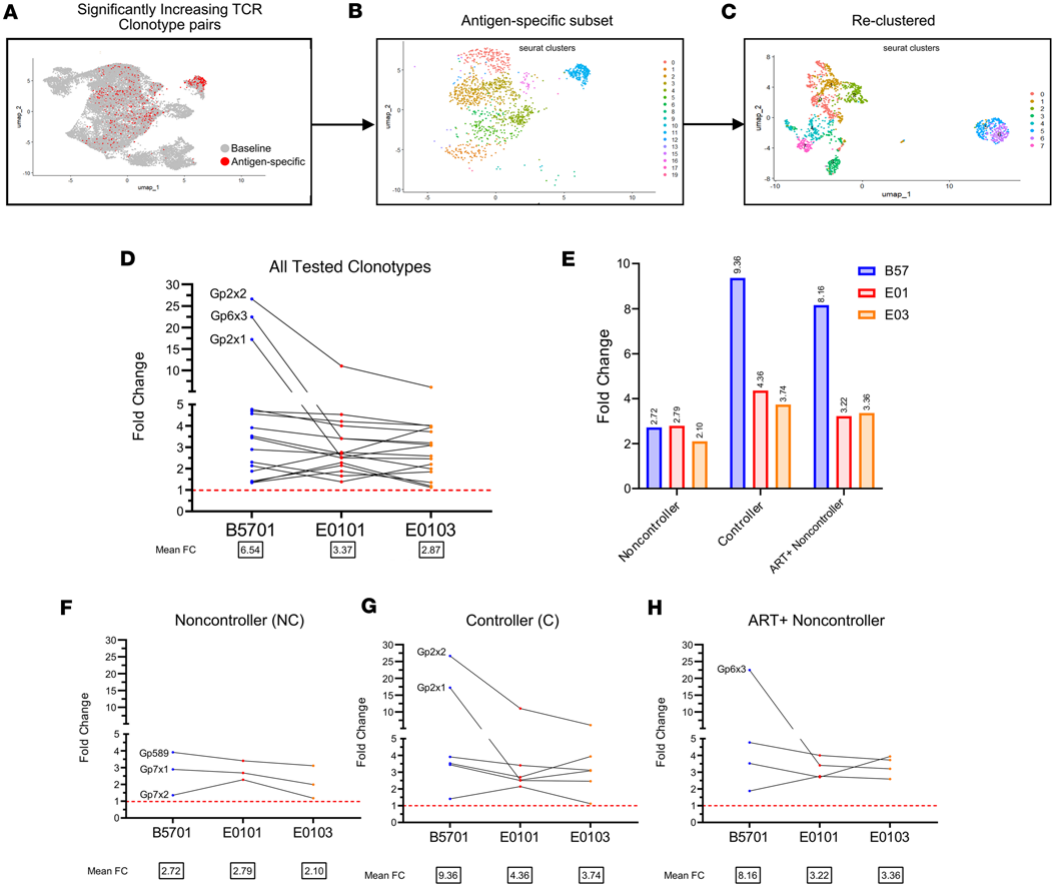
Bioinformatically imputed antigen-specific clonotypes are cross-restricted. (**A**) Distribution of antigen-specific CD8s within full dataset (Figure 4) as identified through VCR-Seek pipeline for cells with paired CDR3 clonotypes found to significantly increase upon stimulation in any metadata combination. (**B**) DimPlot of antigen-specific CD8s from **A**. (**C**) Reclustered antigen-specific subset. Chosen clonotypes identified as being antigen-specific in 10x experiment through described bioinformatics pipeline were cloned into a Jurkat cell line with an NFAT-luciferase reporter. Transformed cells were then cocultured with 41A3.CD4.A02, 41A3.CD4.B57, 41A3.CD4.E01, or 41A3.CD4.E03 cell lines loaded with KF11. (**D** and **E**) The fold change (FC) of all tested clonotypes is shown as a line graph, with the B*57:01, E*01:01, or E*01:03 coculture signal FC of the same clonotype indicated by blue, red, or orange dots, respectively, as luciferase FC over coculture with irrelevant KF11-loaded HLA-A*02–expressing cell line (**D**). The mean FC of all tested clonotypes in the given graphs is shown below the respective condition, and represented as a bar graph for all tested clonotypes split by the control status where the clonotype was found to be statistically antigen-specific through the original bioinformatics pipeline (**E**). (**F**–**H**) Part **A** was then broken down by control status for the non-controller (**F**), controller (**G**), and ART+ NC (**H**). The top 1–3 FC clonotypes for every grouping are indicated next to the B57 dot of the indicated clonotype (“Gp…”).

Traditional TCR analysis does not incorporate a significance testing component. We therefore developed a high-throughput and scalable analysis pipeline to identify all TCR α, β, and paired αβ families or clonotypes that were from the antigen-specific subset, identified as significantly increasing in KF11-stimulated as compared with unstimulated conditions in all metadata contexts (Supplemental Figure 8). When assembled into metaclonotype clusters, antigen-specific TCRs were almost entirely cross-restricted (Supplemental Figure 9). Consistent with our previous results, we again identified a cluster (cluster 6) of TRAV5/TRBV19-encoding cells that were associated with the tri-HLA-restricted phenotype and contained clonotypes overlapping with those identified in the SMART-seq tri-restricted cluster; this cluster was also dominated by clones isolated from the controller and ART+ NC as opposed to ART-naive NC. Extending our earlier observations, cluster 6 also includes TCR sequences isolated from KF11-stimulated PBMCs, indicating that this population was not simply an artifact of using in vitro–generated single HLA-expressing cell lines for antigen presentation but that it can also be observed in the context of an ex vivo response. CD8s from the whole dataset were labeled as “antigen-specific,” i.e., those possessing an antigen-specific TCR (Figure 5A); “subset” (Figure 5B); and then “reclustered” (Figure 5C) for downstream analysis.

These TCRs were again tested for HLA restriction through NFAT reporter assays. All TCRs were cross-restricted, more consistently than TCRs identified through the previous method (Figure 5D). Notably, TCRs from the ART-naive NC had lower TCR affinity across all HLAs (Figure 5E). One clonotype in particular, Gp2x2, was found to increase only in the controller and demonstrated extremely high avidity for KF11 across all 3 HLAs (26-, 11-, and 6-fold change across B*57, E*01:01, and E*01:03, respectively). All other tri-restricted clonotypes demonstrated high but roughly equal avidity across all 3 alleles in comparison with Gp2x2, Gp6x3, and Gp2x1, in contrast to previously isolated TCRs within NC (Figure 5F), C (Figure 5G), and ART+ NC (Figure 5H).

### IL-15 vaccine response signature unique to activated cross-restricted B57/E01-restricted T cell cluster.

**Figure 6:**
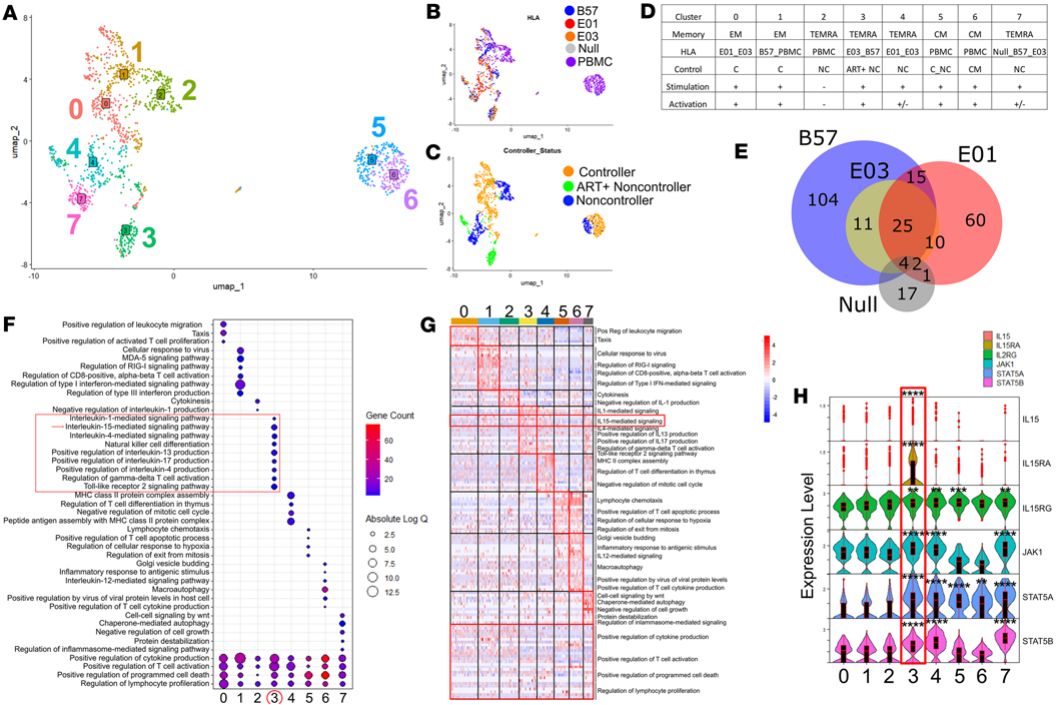
IL-15 vaccine response signature observed in an E03/B57 dually restricted cluster. (**A**) UMAP of Seurat clusters for antigen-specific subset of 10x Genomics dataset. (**B** and **C**) Metadata distribution of HLA (**B**) and controller status (**C**). (**D**) Metadata membership table of each Seurat cluster based on highest relative metadata-labeled cell number in each cluster. Control and activation status were assigned by cell using percentile cutoffs as indicated in Methods. Dual or tri-membership indicates that a similar number of cells from those conditions were represented in the indicated cluster. (**E**) Venn diagram of marker overlap by MAST between B57-, E01-, E03- and null-CD8s. (**F**) Bubble plot showing top Gene Ontology enrichment terms organized as, first, unique to given clusters, then top terms shared by all clusters, colored by marker gene count and sized by FDR [absolute log(*Q*)] value of term. (**G**) Heatmap of top genes included in each term organized by term and cluster. (**H**) Violin plot of top genes within vaccine response signature IL-15 signaling pathway. **P < 0.01, ***P < 0.001, ****P < 0.0001.

Prior data from rhesus macaques showed involvement of IL-15 signaling in MHC-E–restricted SIV-protective responses (13). We therefore examined whether similar signatures appeared in human HLA-E–restricted CD8s. Limiting analysis to significantly antigen-induced TCR clonotypes improved clustering resolution (Figure 6A), resulting in identification of 8 distinct cell subsets (labeled as 0 to 7). Cells clustered more distinctly by HLA restriction (Figure 6B) in this analysis than in the complete dataset, though the separation between clusters was still driven mainly by controller status (Figure 6C). Interestingly, upon deeper examination, clonotypes segregated into distinct clusters that displayed distinct transcriptional signatures (Supplemental Figure 10). This suggests that HIV-specific T cells are functionally distinct from one another at the clonotype level.

Using quantile expression of CITE-seq markers, cells were assigned activation and memory states, and clusters inherited the metadata based on number of cells with each metadata type (Figure 6D). Consistent with our previous secretome findings, when directly compared, E01-CD8s were transcriptionally distinct from B57-CD8s, while E03-CD8s were less distinct (Figure 6E).

Gene Ontology analysis of differentially expressed genes within each cluster revealed IL-15 signaling as uniquely enriched in cross-restricted cluster 3 (TEMRA E03_B57 Stimulated Activated), a feature absent in other KF11-specific subsets (Figure 6F), characterized by a set of immune markers (Figure 6G). Notably, all 6 genes mapping to this pathway were significantly upregulated in this cluster (Figure 6H).

### Ex vivo detection of functionally distinct HLA-E and dually restricted HLA-B*57 KF11 αβ CD8s.

**Figure 7:**
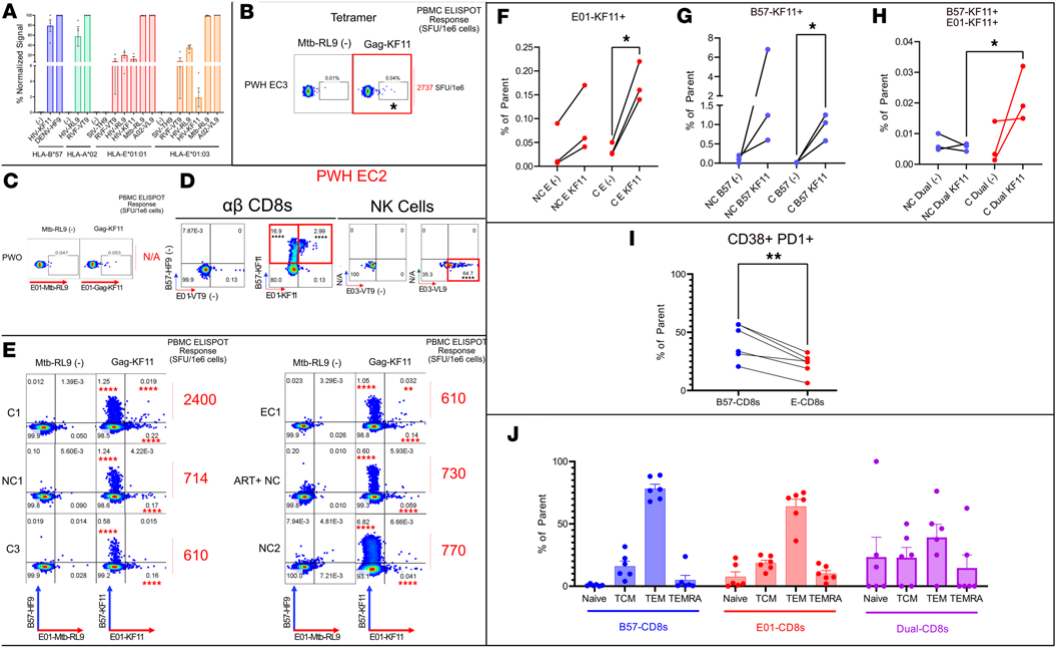
Dually restricted and HLA-E–only–restricted CD8s are observed at higher frequency in controllers. (**A**) In silico exchange or loading rate for indicated peptides normalized to positive control peptide DENV-B*57-HF9, RVF-A*02-VT9, or A*02-VL9 for HLA-B*57, HLA-A*02, or HLA-E*01:01/E*01:03, respectively. (**B**) Ex vivo detection frequency for PWH EC2 to HLA-E*01:01–KF11–PE tetramer. (**C**) Dextramer-PE E-CD8 response rate in a person without HIV (PWO). (**D**) EC2 dextramer-PE/B57 dual restriction response rate to E*01- and B*57-KF11–loaded/exchanged dextramer as well as to VL9 in CD56^+^ NK cells. (**E**) B57- versus E*01-KF11 response rate for leukapheresed PWH compared with Mtb-RL9/B57-HF9 control. ELISPOT response rate to KF11 is shown at right. (**F**–**H**) Summarized single or dual response magnitude (percent of parent), separated by control state, for HLA-E*01:01–KF11–only–, (**G**) HLA-B*57:01–KF11–only–, or (**H**) dually HLA-B*57:01/E*01:01–KF11–only–restricted αβ CD8s from **E** shown as line graphs. (**I**) Summary line graph for CD38^+^PD-1^+^ exhausted B57 or E-CD8 frequency across cohort. (**J**) Summary of T cell memory state frequencies within B57-CD8s (HLA-B*57 restricted), E01-CD8s (HLA-E*01 restricted), and Dual-CD8s (Dual-restricted) across cohort. Gating strategy used for cohort memory states shown in Supplemental Figure 11 using representative PWH EC2. *P* value significance for **B**–**E** was determined by Fisher’s exact *t* test compared with corresponding quadrant or gate of irrelevant peptide negative control. *P* value for **F**–**I** was determined by paired *t* test (if linked line) or unpaired *t* test (if unlinked line, different PWH). *P < 0.05, **P < 0.01, ****P < 0.0001. For **F**–**J**, *n* = 6 from **E**.

Previous E-CD8 detection incorporated co-incubations based on CD8/peptide-pulsed antigen-presenting cells and the use of upregulation of activation markers as surrogates of an antigen-specific response as opposed to assessing direct TCR-HLA interaction. To detect E-CD8s ex vivo circulating in PWH PBMCs, we turned to HLA class I multimer technology. We first tested the ability of KF11 to bind its canonical HLA-B*57 as well as the proposed HLA-E*01:01 and HLA-E*01:03 alleles. HIV-KF11 and HIV-RL9 properly loaded canonical HLA-B*57 and -A*02 monomer (Figure 7A). They both additionally bound HLA-E*01:01. However, HIV-KF11 exchanged into HLA-E*01:03 at half the rate of -E*01:01. Therefore, only HLA-E*01:01–exchanged KF11 was tested alongside -B*57 KF11 in additional assays.

We initially used Gag-KF11 tetramer–PE to test other PWH with more immunogenic (>1,000 SFU per 1 × 10^6^ cells) KF11 responses for ex vivo E-CD8 KF11 restriction, but were only able to identify a modest E-CD8 KF11 population in EC2, an elite controller with a high-magnitude KF11 response in an IFN-γ ELISPOT assay (2,737 SFU per 1 × 10^6^ cells), at 0.04% E-KF11 over 0.01% irrelevant peptide (Figure 7B). To increase sensitivity, we then constructed HLA-E*01:01 and -B*57 dextramers (higher valence), which were not recognized in a person without HIV (PWO) (Figure 7C). We identified a much higher B57-CD8 (16.9% of αβ CD94^–^ CD8s) as well as a dually B57/E-CD8 KF11–specific (2.99% of αβ CD94^–^ CD8s) population ex vivo in that same EC2 through dextramer as compared with tetramer (Figure 7D). A majority of NK cells (64.7%) recognized HLA-E*01–restricted A*02-VL9 over the irrelevant peptide control, validating HLA-E–restricted dextramer specificity (Figure 7D).

Having confirmed dextramer specificity, we then tested the 6 PBMC samples for ex vivo E-CD8, B57-CD8, and dual-CD8 recognition. These were the same PWH samples whose CD8s were sorted via activation-based assays (in vitro) for previous experiments (Figures 1–6). Our dual dextramer staining data demonstrate that all samples showed a detectable E-CD8 response ex vivo at an individual sample level (Fisher’s exact *t* test) (Figure 7E).

When compared between C and NC, this increase in dually staining dextramers was only significant over controls by paired *t* test in the controllers (C1, C3, EC1) (Figure 7F). All PWH also had detectable and higher B57-CD8 recognition of KF11 ranging from 0.58% to 6.82% of αβ CD8s, with the highest in a non-controller (NC2). However, owing to high B*57-only expression observed in NC2, this increase over irrelevant peptide was only significant by paired *t* test in controllers (Figure 7G). The most striking result was that, while weaker than the highly immunodominant response of EC2, significantly positive KF11-specific dually restricted E/B57-CD8 ex vivo detection was only observed in controllers (C1, C3, EC1) and none of the non-controllers (NC1, NC2, ART+ NC), and was significantly higher by magnitude in the controllers (Figure 7H).

To further examine the functional distinction of E- versus B57-CD8s, we examined the exhaustion state of these subsets by dual CD38^+^ and PD-1^+^ staining. Representative gates for CD38^+^PD-1^+^ (Supplemental Figure 11A) or memory subset (Supplemental Figure 11B) staining within E-, B57-, or dual-CD8s within cohort are shown. E-CD8s had lower expression of exhaustion markers compared with B57-CD8s (Figure 7I). Additionally, the same gates for Figure 7D were applied to all the samples shown in Figure 7E, and it was found that across all samples, B57-, E-, and dual-CD8s were enriched for the TEM phenotype, although the latter tended to have less of this phenotype (Figure 7J).

## Discussion

These studies demonstrate primarily that HIV-specific (Gag-KF11) αβ CD8s can cross-recognize HIV antigen across HLA-Ia and HLA-E. This was demonstrated using two approaches — in vitro AIM-based sorting and 10x Genomics platform-based transcriptional analysis — which identified TRAV5 TRBV19 family clonotypes overlapping in B57- and E-CD8s, primarily in controllers. The same clonotypes identified here also overlapped with antigen-specific and high-affinity TCRs previously identified in tetramer-based sorting within the same cohort (14, 15). In those studies, the TRAV5 TRBV19 clonotype was observed to be specific for KF11, though only through HLA-B*57, further confirming the validity of our assays and sorting methods (16). In our work, we determined that the TCRs identified through these different assays all had the capacity to be cross-restricted by HLA-B*57 and HLA-E*01:01 through a Jurkat NFAT-luciferase–based reporter assay, with an enhanced level of cross-restriction observed through the use of our 10x-based VCR-Seek pipeline to identify antigen-specific TCRs. In particular, one clonotype (Gp2x2) was found to have high avidity for KF11 across all 3 HLAs (26-, 11-, and 6-fold change for B57-, E01-, and E03-KF11, respectively). In comparison with controller and ART+ NC clonotypes, the 3 non-controller clonotypes tested demonstrated overall lower avidity for KF11 across all 3 HLA alleles, suggesting lower cross-restriction capacity. However, true dual restriction of the same CD8 requires multimer-based ex vivo detection, which we developed. Upon performing of these assays, this dual restriction was confirmed only in controllers and elite controllers. Counterintuitively, ex vivo dual restriction displayed relatively low frequency only among controllers, despite the high number of in vitro–activated TCRs confirmed to have dual affinity through Jurkat screening. This disconnect may be explained when considering that only a few TCRs displayed extremely high cross-restricted affinity (such as Gp2x2), which may suggest that only the highest-avidity (and most antigen-specific) CD8s are cross-restricted.

Despite the small sample size, our data suggest that in some controllers there is cross-recognition of HIV-1 across HLA-Ia and -E. It is known that HIV-1 downregulates HLA-Ia through mechanisms mediated by Nef and perhaps Vpu (17–19). This process has been well characterized as a way for the virus to escape and evade T cell–mediated immune responses. As the founder virus adapts and downregulates HLA-Ia in an infected individual, HLA-E expression remains unaffected. This may therefore allow HLA-E to present the same epitope to the subset of CD8s that would recognize HLA-Ia–restricted HIV-1 epitopes. Although the primary purpose of continued HLA-E expression on an HIV-infected cell is to evade the innate NK cell clearance (20), it may also contribute to maintaining the adaptive CD8^+^ T cell response.

A combination of low affinity of HLA-E for the peptide and low affinity of the KF11/HLA-E complex for the TCR (21–25) could help explain the functional differences that were observed when HLA-E–restricted and HLA-B57–restricted CD8^+^ T cell responses were compared,\ though some unique cytokines were found to be enriched in E-CD8s (IL-27, RANTES, IP-10). Although HLA-E–restricted CD8^+^ T cell responses were low in frequency, they were consistently detected in all 7 PWH who exhibited positive KF11-specific responses by ELISPOT. Notably, dually restricted responses were observed in 3 of these 7 individuals. Thus, despite the small sample size, our ability to identify such responses ex vivo via dextramer staining represents an important finding in the context of PWH, as most prior HIV-specific HLA-E responses have been studied using CD8s primed from naive donors. Thus, our data provide a foundational framework for future identification of HLA-E–restricted CD8s specific for new epitopes in PWH using assays and reagents recently developed by our group and others (5, 6, 26–28).

A small sample size precludes our ability to correlate targeting of HLA-E–restricted CD8s and viral control, and future studies in larger PWH cohorts are needed to determine the functional significance of HLA-E–restricted low-avidity CD8s. Prior studies have shown that despite low avidity, CD8^+^ T cell responses induced in response to classical HLA-Ia alleles can play an important role. Specifically, such T cell responses, often subdominant, can respond to antigen stimulation, proliferate, support the expansion of higher-affinity CD8s, and recognize a broader range of viral variants, to mitigate viral escape (29–33).

To date, HIV-specific E-CD8s have been described for 4 epitopes (3 in Gag and 1 in Rev) (5, 6, 34), so these responses may appear to be rare in PWH. However, these epitopes were identified based on labor-intensive detection methods such as coculture-based assays and CD8^+^ T cell priming from HIV-naive donors. Large-scale studies to detect E-CD8 responses to these 4 epitopes across a PWH cohort with varying levels of viral control off ART have not been performed to determine their prevalence and biological relevance, as these epitopes generally have much lower affinity for HLA-E than VL9 (9, 21, 22, 24, 28). Use of approaches to enhance peptide/HLA (pHLA) and pHLA/TCR avidity interaction (35–40) will allow more comprehensive studies to be performed on pathogen-specific E-CD8s. Similarly, recent advances in the field (5, 6, 26–28, 41) in the refining of peptide motifs for HLA-E epitopes, HLA-E binding predictions, use of cell-based stabilization assays, and the development of monomer binding–based multimer assays have the potential to improve the identification and characterization of new epitope-specific E-CD8s in the context of HIV infection. Considering the low magnitude of HLA-E responses observed in our data, future studies examining functional aspects of E-CD8 responses may also be greatly improved by the use of single-cell-based cloning of antigen-specific E-CD8s in combination with dextramer staining–based sorting to enrich for high-magnitude responses. Despite our small cohort, the fact that 7 of 7 tested PWH with an immunogenic response to KF11 demonstrated E-CD8 recognition if KF11 ex vivo also suggests that with an assay based on more sensitive dual HLA class I detection, HIV-1-specific E-CD8s may be more common than previously assumed. Our results based on a single epitope examined in a cohort with a small sample size limit the generalizability of our study. Nevertheless, CD8s capable of recognizing the same peptide presented by HLA-B*57 and HLA-E represent a finding with important implications for T cell immunity to a variety of pathogens besides HIV.

We also found that in each HLA-restricted context, E- and B57-CD8s demonstrate distinct functionality at the secretomic (Luminex), transcriptional (scRNA-seq), and cell surface marker (flow cytometry) levels. In our study, RANTES, IP-10, and IL-27 were observed to increase in an HLA-E–restricted and antigen-specific manner, while the more classical CD8 cytokines were observed in the B57-specific response, including IFN-γ, TNF-α, MIG, IL-6, MIP-1β, IL-2, and IL-9. In addition, 2 of 3 E-CD8 and 4 of 7 B57-CD8 cytokines were confirmed to be transcribed by the appropriate HLA-restricted CD8 subsets in coculture assays based on scRNA-seq data. Although previous studies have shown that transcriptomic and proteomic data can often be discordant as a result of differences in assay methodologies and cellular activation states (42–44), many of our proteomic findings (2 of 3 E-CD8 and 4 of 7 B57-CD8 cytokines observed to be secreted) also exhibited elevated expression at the transcriptional level. This demonstrates a notable concordance between data obtained from 2 distinct assays. E-CD8s also demonstrated a lower exhaustion profile than B57-CD8s in the context of KF11-specific responses, consistent with this distinction. Lastly, multiply-restricted activated CD8s identified in in vitro experiments were found to have higher metabolic transcriptional signatures by Gene Ontology analysis, similarly consistent with the finding that they are functionally distinct despite their cross-restriction. However, both E- and B57-CD8s are primarily TEM or TEMRA, consistent with previous studies on the memory state of E-CD8s in CMV and Mtb (45–47) and supporting the finding that E-CD8s and B57-CD8s are an overlapping subset of CD8s specific for the same antigens. Also consistent with previous studies was the secretomic profile of the cytokine classes observed from E-CD8s. While they were mainly observed to secrete IL-27 and RANTES (antiviral chemokines), they secreted various other non-cytotoxic and helper cytokines alongside B57-CD8s, such as IL-2, which has been previously documented for E-CD8s (48, 49).

Study of cross-restricted CD8^+^ T cell priming is crucial in the design of an effective HIV-1 vaccine. A recent study on clonotypes induced by an Ad5 HIV vaccine closely examined the repertoire of 3 B57 elite controllers/long-term non-progressors (EC/LTNPs) and 3 B57 vaccine recipients (50). Three of the four clonotypes obtained from the EC/LTNP group included TRAV5-TRBV6-1 and TRAV5-TRBV7-9, which map to our clusters 5 and 6, the “tri-restricted” clusters identified in controllers and the ART+ NC. However, the dominant clonotype identified in the HIV vaccine recipients was TRBV20-1, which belongs to cluster 1, an ART-naive non-controller cluster in our current study. This suggests a potential reason why prior vaccine trials that induced CD8s failed to impact infection or disease progression, as they appeared to elicit clonotypes associated with ART-naive non-controllers. Furthermore, this study indicated that the vaccine drove insufficient clonal selection, potentially eliciting the “wrong” antigen-specific clonotypes associated with non-controllers, rather than the tri-restricted, efficacious CD8^+^ TCR clonotypes identified here as associated with control. Additionally, the main finding of that study was that the TCRs elicited in vaccinated participants had low affinity. Our current findings support this, suggesting that these low-affinity CD8s are unlikely to cross-recognize epitopes presented by HLA-E, which may have negatively affected vaccine efficacy. Our in vitro assays further confirmed that the TCRs identified from the non-controllers also exhibited similarly low affinity.

Prior work based on bulk RNA sequencing analysis showed that in the context of SIV vaccine–induced MHC-E-CD8s, an IL-15 response uniquely distinguished macaques in which the establishment of infection was prevented (13). Among antigen-specific CD8s specific for KF11, we similarly observed a cluster of activated, antigen-specific dually restricted E03/B57-CD8s demonstrating a similar IL-15 signaling pathway transcriptional signature. In the previous study it was not possible to determine which cell types were responsible for secretion of IL-15. Here, we determined that this transcriptional signature emanated from dually restricted B57- and E-restricted CD8s that were both responding to (IL15RA, various downstream TCR stimulation markers) and producing IL-15.

To the best of our knowledge, our study is the first to identify ex vivo–based E-CD8 responses in PWH by using multimer-based technology as well as to detect dually HLA-B57– and -E–restricted CD8s. However, the question remains of whether observed differences in functionality are due to weaker TCR signaling on E-CD8s in comparison with B57-CD8s. First, while the frequency of E-CD8s observed ex vivo may be relatively low, prior studies have shown that they can secrete a range of cytotoxic and helper cytokines, including IFN-γ, TNF-α, IL-2, IL-4, and IL-13, which can promote proliferation and regulation of B and T cell subsets in a Th2-like capacity (47, 49, 51). Consistent with these reports, we identified IL-27 and RANTES expression in E-CD8s, both of which have demonstrated antiviral properties against HIV-1 through coreceptor blockade or inhibition of virion production and cell-to-cell spread (52, 53). Together, these observations suggest that HLA-E–restricted responses, while not necessarily functioning as primary cytotoxic effectors, may play complementary regulatory and antiviral roles that contribute to the overall immune response to HIV. Future studies aimed at further elucidating the role of HLA-E–restricted CD8s could incorporate approaches such as the generation of CTL clones or the use of dextramer-sorted cells, which would provide cleaner readouts by minimizing potential confounders in functional assays. The application of tagged multimers, including dextramer technology, in combination with single-cell transcriptional and functional analyses will allow a more rigorous assessment of mono- and dual-restricted TCRs in larger cohorts. The analytical pipelines and molecular techniques developed here will therefore enable future studies aimed at determining the prevalence of E-CD8s and dual-CD8s within different states of viral control.

## Methods

Further information can be found in Supplemental Methods.

### Sex as a biological variable

Sex was not considered as a biological variable in this study owing to limited sample availability.

### Study cohort

All participants were recruited at Vanderbilt University, Nashville, Tennessee. PBMCs were collected by leukapheresis from 8 people living with HIV (PWH) who expressed HLA-B*57. HIV clinical status was determined based on plasma viral load at the time of sample acquisition (>2,000 RNA copies/mL for non-controllers [NC], <2,000 copies/mL for controllers [C], and <50 copies/mL for elite controllers [EC], and one ART+ NC). The clinical and HLA genotyping data are shown in Supplemental Table 1.

### Cell lines

Parental 721.221 or transduced 721.221 cell lines, a gift from Jun Kan-Mitchell (University of Texas at El Paso, El Paso, Texas, USA), first had HLA-E expression knocked out at a site in the first exon to generate the 41A3 cell line, before transduction of CD4 and either HLA-B*57:01, HLA-E*01:01, or HLA-E*01:03 to generate the 41A3.CD4.[B57, E01, or E03] cell lines (5). These cell lines were cultured in R-20 (20% FBS in RPMI 1640 supplemented with penicillin/streptomycin, l-glutamine, HEPES, and sodium pyruvate) and assessed for cell surface HLA expression of the various HLA molecules and CD4 via flow cytometry.

### Peptide

The KF11 peptide (KAFSPEVIPMF, Gag [162–172]) as well as other control peptides DENV-HF9 (HTWTEQYKF, NS1 [26–34]), RVF-VT9 (VLSEWLPVT, N [121–129]), HIV-RL9 (RMYSPTSIL, Gag [275–283]), Mtb-RL9 (RLPAKAPLL, InhA [53–61]), SIV-TH9 (TVCVIWCIH, Gag [33–41]), and human A*02-VL9 (VMAPRTLVL, HLA-A*02 leader peptide) were purchased from Genscript, reconstituted in 100% DMSO, and stored at –80°C until use. HIV-1 Gag, Nef, and Pol overlapping peptide pools were obtained from the NIH AIDS Reagent Program (catalog ARP-8117, -12545, and -12438, respectively). Peptide pools were used at 1 μg/mL and single peptide was used at 10 μg/mL, unless otherwise specified. Single peptides used for monomer and multimer assays were used at 100 mg/mL.

### IFN-γ ELISPOT

IFN-γ enzyme-linked immunosorbent spot-forming assay (ELISPOT) screening of samples for previously described HLA-B*57–restricted HIV-1 epitopes including KF11 was performed as previously described (5, 54, 55). In brief, cryopreserved samples were thawed in R-10 medium (RPMI 1640 base plus streptavidin/penicillin, l-glutamine, and HEPES), and PBMCs were allowed to rest at 37°C overnight. Nitrocellulose plates (MilliporeSigma) were coated with 10 μg/mL anti–IFN-γ monoclonal antibody overnight at 4°C, and excess uncoated antibody was washed off before wells were blocked with 200 μL R-10 for 2 hours at 37°C. R-10 was discarded, and then PBMCs were plated in duplicate wells at 100,000 cells per well in 100 μL each before antigen was added in 100 μL for a final concentration of 10 μg/mL of single antigen per well. PBMCs incubated with no antigen or phytohemagglutinin were used as negative and positive controls, respectively. After about 18–24 hours, wells were washed and incubated with biotinylated anti–IFN-γ antibody for 2 hours, washed again and incubated with streptavidin–alkaline phosphatase for 1 hour, then developed with NBT/BCIP substrate for 5–10 minutes. The CTL ImmunoSpot analyzer (version 5; CTL) was used to count raw spots per 1 × 10^5^ cells, which was normalized to spots per 1 × 10^6^ cells (SFU per 1 × 10^6^ cells). A positive response was defined as one that was at least 55 SFU per 1 × 10^6^ cells and 4 times background (DMSO).

### Coculture assay

The dual CD69/CD137-positive response to HLA-expressing lines pulsed with KF11 was compared with that of the HLA-null parental line (721.221ΔE.E) pulsed with KF11 (Supplemental Figure 3, A and B). The activated population was compared in the CD4/14/16/19/56^–^, CD8^+^ CD3-dim population, as CD8^+^ T cells downregulating CD3 and CD8 coreceptors have been shown to be more antigen specific and higher-avidity (56–60), and thus are a better indicator of an HLA-restricted antigen-specific response in the cell line–dependent co-incubation system where nonspecific activation is commonly observed. Those donors who demonstrated a CD69^+^CD137^+^ response in this subset that was 3 times over the 41A3.CD4 null line response and significant by Fisher’s exact *t* test (*P* < 0.0001) were identified as demonstrating a high-avidity HLA-restricted response to KF11.

#### Ex vivo activation–based single-cell sorting and 96-well plate scRNA/TCR sequencing.

Assays were done as previously described (5) with the following alterations. Initially, the specified cell lines (41A3.CD4, 41A3.CD4.B57, 41A3.CD4.E01, 41A3.CD4.E03) were pulsed with KF11 peptide at 10 μg/mL for 2 hours at 37°C before peptide was washed off with 1× serum-free medium. CD8s were then isolated from PBMCs using the StemCell Easy-Sep CD8 Enrichment kit (catalog NC0050243) and co-incubated with peptide-pulsed cell lines as indicated for 18 hours in the presence of anti-CD28 and anti-CD49d. Cells were then stained with the sorting antibody mix (Supplemental Table 2). Per the gating strategy shown in Supplemental Figure 1A, single-cell lymphocytes were gated on, followed by live cells (aqua^–^), CD4/dump^–/–^, CD8^+^ CD3-dim, and CD69^+^CD137^+^. Cells that fit this gating strategy and met the criteria of having a CD3-dim activated response to the given HLA pulsed with KF11 significantly (and 3 times) higher than that of 41A3.CD4 cells also pulsed with KF11 were then sorted into a 96-well plate containing the lysis buffer previously described using the FACSAria II cell sorter (BD Biosciences). scTCR-seq and scRNA-seq were performed as previously described (5, 55, 61, 62).

#### 10x sample preparation.

CD8s from 3 PWH, one from each control status (*n* = 1 NC, ART+ NC, and C) of the previous *n* = 8 cohort, were cocultured with CD8s as described for that set of experiments. These cocultures were performed in wells of a 24-well plate per instructions of the Flow Cytometry and Single Cell Core at the University of Alabama at Birmingham. After 18 hours of co-incubation, cells were washed and first stained with Hashing antibodies 1–10 (C0251–C0260) from BioLegend (catalog number of C0251 is 394661 for reference to set) per manufacturer instructions after initial titration of both hashtagging antibodies (data not shown). After washing off, cells were stained using the same panel as previously outlined in addition to the following CITE-seq antibodies with the exact same staining protocol: TotalSeq-C anti-CD3, -CD4, -CD8, -TCRαβ, -CD56, -CD69, -CD137, -CD107a, -PE, -APC, -FITC, -CD45RA, -CD45RO, and -CCR7. Unactivated (CD69^–^CD137^–^) CD56^–^ CD8^+^ cells from the unstimulated CD8s (no KF11 loading for 41A3 lines or pulse for PBMCs) as well as both unactivated and activated CD8s from stimulated conditions were sorted using the BD Biosciences FACSymphony S6 cell sorter into a single tube, and multiplexed for all 10 samples for 1 of the 3 PWH per day. Libraries from these cells were then generated on that same day for RNA expression (gene expression), TCR, and CITE-seq, and then these separate hashtagged libraries were all sequenced on the Illumina NovaSeq using an S1 flow cell to cover 1.6 billion reads across the estimated 22,172 cells sorted after manual quality control before library generation. Specifically, cell suspension, 10x Genomics barcoded gel beads, and oil were loaded into 10x Genomics Chromium Single Cell Chip K (PN-1000287). Chromium Next GEM Single Cell 5′ v2 Kit (PN-1000263) was used to capture single cells in nanoliter-scale oil droplets by 10x Genomics Chromium Controller and generate Gel Bead-In-Emulsions (GEMs). The 5′-biased gene expression, TCR, and feature barcoding libraries were generated following the manufacturer’s instruction. All libraries were sequenced by an Illumina NovaSeq 6000 machine, targeting 20,000 read pairs per cell for gene expression libraries, 20,000 per cell for TCR libraries, and 5,000 per cell for feature barcoding libraries; the sequencing cycles consisted of 26 bp for read 1, 90 bp for read 2, and 10 bp for i7 and i5.

### TCR clustering analysis

Initial clustering analysis was performed as previously described (5), except that the newest release of TCRdist (TCRdist3) was used (https://github.com/kmayerb/tcrdist3) (63). TCRdist performs pairwise assessments of TCR sequences based on their amino acid similarity in complementarity-determining regions (CDRs) 1, 2, and 3. Initial Hamming distances were calculated and distance matrices were generated using the available Python package in R (reticulate package, https://rstudio.github.io/reticulate/). Distance matrices for the complete dataset (with associated metadata) were visualized using Cytoscape (v3.10) (64). Metadata linked with individual TCR clonotypes were used to associate each clonotype (or cluster) with HLA restriction, HIV control status, or stimulation state based on other analyses.

### Luminex assay and analysis

The Luminex assay was performed using the MagPix instrument running xPONENT software (Luminex Corp.) in collaboration with Davide Botte of the Lund lab (University of Alabama at Birmingham, Birmingham, Alabama, USA). Readouts were analyzed with EMB Millipore’s Milliplex Analyst Software. A custom 38-plex human cytokine/chemokine panel was used for this assay (catalog HCYTA-60K-PK38) for the following cytokines: EGF, CCL11, G-CSF, GM-CSF, IFN-α2, IFN-γ, IL-1α, IL-1β, IL-1RA, IL-2, IL-3, IL-4, IL-5, IL-6, IL-7, IL-8, IL-10, IL-12 p40, IL-12 p70, IL-13, IL-15, IL-17A, IL-17E, IL-17F, IL-18, IL-22, IP-10, MCP-1, M-CSF, MIG, MIP-1α, MIP-1β, PDGF-AA, PDGF-AB/BB, RANTES, TNF-α, LTA, VEGF-A. The samples assayed were 200 μL supernatants from HLA-null or -overexpressed 41A3.CD4 cell lines loaded with either no peptide or KF11 and cocultured with isolated CD8s from leukapheresis samples of PWH at various control states. The supernatants were initially spun down to exclude cell debris and then stored at –80°C until the time of the assay. Initially, raw data were quality-controlled, and any cytokine with values outside the standard curves obtained per manufacturer recommendation were excluded (only IL-17F, which was below the limit of detection). Raw MFI signal values for each sample for each cytokine were compared with the standard curves to generate pg/mL values through the proprietary analysis software Quantist (Bio-techne; https://www.bio-techne.com/reagents/luminex/luminex-software-quantist). Values for each donor sample were then normalized to the 41A3.CD4-KF11 pulse coculture condition value of that sample as the optimal negative control for a non-HLA-restricted non-allogeneic response. These normalized values were then analyzed initially through GraphPad Prism for direct pairwise comparisons. All response magnitudes were then visualized through JMP (SAS Institute Inc.) alongside statistically significant pairwise ANOVA comparisons. To analyze these data, a 2-way ANOVA was first performed on both the unstimulated and HLA-KF11 response replicates between HLA-B*57 and -E*01:01 or -E*01:03 as normalized to HLA-null line–KF11 background pg/mL. Those cytokines for which HLA restriction as a categorical variable explained a significant (*P* < 0.05) amount of the quantitative variation in concentration of the listed cytokine (pg/mL) in the 2-way ANOVA or which demonstrated a significant increase in only HLA-B*57 or E*01:01/03 conditions were identified. Determinant analysis and canonical clustering were also performed through JMP using available metadata under “Analysis” > “Multivariate methods” > “Discriminant,” with each individual cytokine for all HLA-dependent conditions given as a covariate numerical value and grouped by that condition. Individual cytokines that were shown to demonstrate a significant increase in response between HLA-B57 and either E condition were then represented through Prism.

### TCR reporter cell assay

The in vitro function of selected TCR clones was assessed using a luciferase reporter cell assay, as described previously (10). Briefly, TCR α and β sequences obtained from single-cell data were reconstructed as full-length, codon-optimized cDNA products and synthesized as gBlocks gene fragments (Integrated DNA Technologies). TCR genes were cloned into the eukaryotic expression vector, pSELECT-GFPzeo (InVivoGen). Jurkat effector T cells were prepared by cotransfection with plasmids encoding the TCR α/β clone of interest along with a plasmid expressing the CD8α protein (pORF9-hCD8A, InVivoGen) and a reporter plasmid encoding firefly luciferase driven by a minimal nuclear factor of activated T cells–responsive (NFAT-responsive) promoter (pNFAT-luc, Agilent Technologies). Target cells expressing single HLA molecules (41A3.CD4.[B57, E01, or E03], described above) were pulsed with KF11 peptide (10 μM), or left as unpulsed controls, and then cocultured with Jurkat/TCR^+^ effector cells at an effector/target cell ratio of 2:1. After 6–8 hours, the luciferase activity in cocultures was measured using the SteadyGlo reagent (Promega).

### Peptide exchange and ELISA

7MT2 UV-labile VL9 “J” VMAPJTLVL peptide–loaded pHLA-E monomer was provided by the NIH Tetramer Core Facility (NIH Contract 75N93020D00005 and RRID:SCR_026557) located at Emory University, Atlanta, Georgia. Peptide exchange was performed in a modified form from what has been previously described (9, 22, 23, 27). Following various optimization experiments, the protocol for this assay was optimized as follows. Initially, ELISA high-binding plates were coated with 10 μg/mL purified anti–HLA-E antibody (3D12, catalog 342602, Biolegend) overnight at 4°C in coating buffer (catalog NC0130210, Biolegend). After initial overnight coating at 4°C, another overnight blocking with 2% IgG BSA (catalog 12-662-550ML, MilliporeSigma) was performed. The following day, peptide exchange was performed in wells of a non-binding V-bottom plate covered with a plate heat shield cover. Each 125 μL reaction contained 121.5 μL of peptide exchange buffer, 1.5 μL stock monomer (3 μg 7MT2, stored at 80°C), and 2 μL query peptide (200 μg). Peptide exchange buffer stock was generated using 1 mL 1 M Tris-HCl (pH 8) (catalog AAJ22638AE, Thermo Scientific), 842.64 mg l-arginine monohydrochloride (11-101-2479, MilliporeSigma), 40 μL 0.5 M EDTA (MT-46034CI, Corning), 15.4 mg reduced glutathione (AC120000050, Thermo Scientific), 3 mg oxidized glutathione (MP021511935, MP Biomedicals), 8.1 mL ddH_2_O, and one peptidase inhibitor cOmplete EDTA-free tablet (11873580001, Sigma Aldrich). Each reaction was gently mixed with a pipette and then UV-irradiated (365 nm) for 3 hours at room temperature. The coated/blocked ELISA plate was then washed as previously described (9) and incubated with 50 μL (1:100) of peptide-exchanged reaction mix for 1 hour. This was carried out at 37°C as previously described to enhance binding and destabilize low-affinity interactions (65). After washing, 50 μL primary antibody (1:500; anti-β2M, catalog R0202-1D, Thermo Scientific) was incubated on the plate for 30 minutes at 4°C, then, after another round of washing, 50 μL secondary antibody (1:500; anti-rabbit–HRP, catalog NB7160, Novus Biologicals) for 15 minutes at room temperature in the dark. Finally, the plate was washed again, and 100 μL TMB substrate (catalog PI37574, Thermo Scientific) was added for 10 minutes (or until sufficient development) before addition of 100 μL ELISA Stop Solution (catalog PIN600, Thermo Scientific). Corrected A450 values (– background A650) were transformed as follows. TMB substrate and Stop Solution was added by row, and so corrected A450 values for each row were normalized separately between 0 [7MT2 (– control) no peptide condition] and 1 [A*02 VL9 (+ control) peptide exchange] with the following calculation:


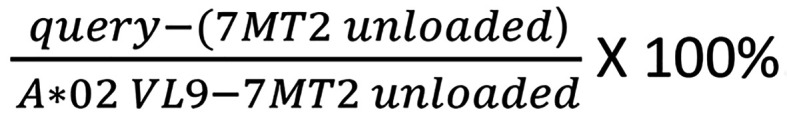


For HLA-A*02 and HLA-B*57, DENV-HF9 (Dengue virus HF9) and RVF-VT9 (Rift Valley fever virus VT9) were used as irrelevant (+) control peptides known to bind HLA-B*57 and -A*02, respectively, and as (–) controls for TCR specificity in a cohort in a region to which Dengue virus and Rift Valley fever are not endemic. We additionally used HLA-A*02–derived VL9 peptide as the positive control for HLA-E*01:01 or -E*01:03 monomer exchange rate.

### Multimer construction

Again, 7MT2 UV-labile VL9 “J” VMAPJTLVL peptide–loaded pHLA-E monomer was provided by the NIH Tetramer Core Facility (NIH Contract 75N93020D00005 and RRID:SCR_026557) located at Emory University. Stable post–peptide exchange pHLA-E monomers were tetramerized per NIH Tetramer Core Facility general guidelines. An unbiased saturation strategy was used. Specifically, immediately after results of ELISA or the following day, 50 μL of reaction mix was transferred to two 1.5 mL microfuge tubes. 2.4 μL or 1.3 μL of 0.2 mg/mL streptavidin-PE or -APC, respectively, was added to either tube and the process repeated 5 times every 10 minutes, to fully saturate any available stable pHLA-E monomer. Alternatively, dextramer was assembled per Immudex dextramer construction guidelines. PE and APC Klickmers used for initial testing and optimization were provided by Immudex ApS. HLA-A*02 and HLA-B*57 monomers used for initial testing were also provided by ImmunAware ApS.

### Multimer staining

Multimer staining (both tetramers and dextramers) was performed as recently described for HLA-E–restricted SARS-CoV-2 (27). In brief, PBMCs were thawed as previously described (5) and incubated with 1 μL per test Fc Block (BD Biosciences) (to prevent FcR-mediated binding) and 1 μL per test dasatinib (to make TCR more bioavailable for recognition) for 5 minutes before washing off. Tetramer or dextramer staining was then performed for 3 hours at room temperature in the dark. For each pool in the case of dual HLA-E*01– and HLA-B*57–restricted multimer staining, 2 μL of 20 μM d-biotin was also added during pool construction to prevent nonspecific streptavidin binding. After another 3 washes, cells were then stained with the multimer staining mix (Supplemental Table 3).

#### Tetramer staining.

PE and APC tetramers were generated and used to stain at 1:100 dilution in FACS-Wash (made in-house; PBS: Corning, FBS, GeminiBio) as described above.

#### Dextramer staining.

PE Klickmers (catalog NC1685800) were generously provided by Immudex ApS for initial optimizations and tests. Before dextramerization, a peptide exchange was performed. The amount of monomer to add was calculated for PE Klickmer based on estimation of 20 acceptor sites filled and micromolarity of pHLA-E-Mtb14 (structure 7P49) (23). One hundred microliters of diluted dextramers was added immediately after dextramerization per Immudex protocol.

#### Klickmer PE.

Staining of samples was performed as described above.

### Statistics

Luminex pairwise 2-way ANOVA and 2-tailed *t* test significance testing as well as Fisher’s exact tests used for flow cytometry analysis were performed using GraphPad Prism version 8.0 software. Differentially expressed gene significance testing was performed using the MAST test through Seurat as described in the package documentation.

### Study approval

This study was approved by the Vanderbilt University Medical Institutional Review Board, and all subjects provided written informed consent (IRB 030005).

### Data availability

All transcriptomic and CITE-seq proteomic data were deposited in the NCBI’s Gene Expression Omnibus database (GEO GSE306022 and GSE306023). Specific analysis files and other data are available upon request. All supporting data shown in graphs are available in the Supporting Data Values file.

## Author contributions

AB, PAG, and KJM conceptualized and designed the study. KJM, AKO, HSH, ZD, MAR, AC, and RW conducted experiments and/or curated data. KJM conducted formal data analysis and statistics. SAK provided clinical specimens. PAG and AB acquired funding. PAG, AB, and MAB supervised the study. KJM, AB, and PAG wrote the manuscript. All authors reviewed and edited the manuscript.

## Funding support

This work is the result of NIH funding, in whole or in part, and is subject to the NIH Public Access Policy. Through acceptance of this federal funding, the NIH has been given a right to make the work publicly available in PubMed Central.

- NIH grant R01-AI162168-04 (to AB and PAG).
- University of Alabama at Birmingham (UAB) Center For AIDS Research, an NIH-funded program (P30-AI027767).
- Supported by the NIH through the T32 training program (T32-AI7051-47).

## Supplementary Material

Supplemental data

Supporting data values

## Version 1 12/18/2025

In-Press Preview

## Version 2 02/09/2026

Electronic publication

## References

[1] Hansen SG (2022). Myeloid cell tropism enables MHC-E-restricted CD8^+^ T cell priming and vaccine efficacy by the RhCMV/SIV vaccine. Sci Immunol.

[2] Hansen SG (2016). Broadly targeted CD8^+^ T cell responses restricted by major histocompatibility complex E. Science.

[3] Voogd L (2022). Antigen presentation by MHC-E: a putative target for vaccination?. Trends Immunol.

[4] Sharpe HR (2019). HLA-E: exploiting pathogen-host interactions for vaccine development. Clin Exp Immunol.

[5] Bansal A (2021). HLA-E-restricted HIV-1-specific CD8^+^ T cell responses in natural infection. J Clin Invest.

[6] Yang H (2021). HLA-E-restricted, Gag-specific CD8^+^ T cells can suppress HIV-1 infection, offering vaccine opportunities. Sci Immunol.

[7] Wallace Z (2024). Instability of the HLA-E peptidome of HIV presents a major barrier to therapeutic targeting. Mol Ther.

[8] He W (2023). Intracellular trafficking of HLA-E and its regulation. J Exp Med.

[9] Walters LC (2020). Detailed and atypical HLA-E peptide binding motifs revealed by a novel peptide exchange binding assay. Eur J Immunol.

[10] Anmole G (2015). A robust and scalable TCR-based reporter cell assay to measure HIV-1 Nef-mediated T cell immune evasion. J Immunol Methods.

[11] Dash P (2017). Quantifiable predictive features define epitope-specific T cell receptor repertoires. Nature.

[12] Finak G (2015). MAST: a flexible statistical framework for assessing transcriptional changes and characterizing heterogeneity in single-cell RNA sequencing data. Genome Biol.

[13] Barrenäs F (2021). Interleukin-15 response signature predicts RhCMV/SIV vaccine efficacy. PLoS Pathog.

[14] Simons BC (2008). Despite biased TRBV gene usage against a dominant HLA B57-restricted epitope, TCR diversity can provide recognition of circulating epitope variants. J Immunol.

[15] Pilkinton MA (2021). In chronic infection, HIV gag-specific CD4^+^ T cell receptor diversity is higher than CD8^+^ T cell receptor diversity and is associated with less HIV quasispecies diversity. J Virol.

[16] Yu XG (2007). Mutually exclusive T-cell receptor induction and differential susceptibility to human immunodeficiency virus type 1 mutational escape associated with a two-amino-acid difference between HLA class I subtypes. J Virol.

[17] Ende Z (2018). HLA class I downregulation by HIV-1 variants from subtype C transmission pairs. J Virol.

[18] Apps R (2016). HIV-1 Vpu mediates HLA-C downregulation. Cell Host Microbe.

[19] Mwimanzi F (2020). An HIV-1 Nef genotype that diminishes immune control mediated by protective human leucocyte antigen alleles. AIDS.

[20] Romero-Martín L (2022). Disruption of the HLA-E/NKG2X axis is associated with uncontrolled HIV infections. Front Immunol.

[21] Huisman BD (2023). High-throughput characterization of HLA-E-presented CD94/NKG2x ligands reveals peptides which modulate NK cell activation. Nat Commun.

[22] Walters LC (2018). Pathogen-derived HLA-E bound epitopes reveal broad primary anchor pocket tolerability and conformationally malleable peptide binding. Nat Commun.

[23] Walters LC (2022). Primary and secondary functions of HLA-E are determined by stability and conformation of the peptide-bound complexes. Cell Rep.

[24] Barber C (2022). Structure-guided stabilization of pathogen-derived peptide-HLA-E complexes using non-natural amino acids conserves native TCR recognition. Eur J Immunol.

[25] Iyer RF (2024). CD8^+^ T cell targeting of tumor antigens presented by HLA-E. Sci Adv.

[26] Vaurs J (2021). A novel and efficient approach to high-throughput production of HLA-E/peptide monomer for T-cell epitope screening. Sci Rep.

[27] Yang H (2023). HLA-E-restricted SARS-CoV-2-specific T cells from convalescent COVID-19 patients suppress virus replication despite HLA class Ia down-regulation. Sci Immunol.

[28] Voogd L (2025). HLA-E/peptide complexes differentially interact with NKG2A/CD94 and T cell receptors. J Immunol.

[29] Frahm N (2006). Control of human immunodeficiency virus replication by cytotoxic T lymphocytes targeting subdominant epitopes. Nat Immunol.

[30] Straub A (2023). Recruitment of epitope-specific T cell clones with a low-avidity threshold supports efficacy against mutational escape upon re-infection. Immunity.

[31] Wang P (2024). Low-affinity CD8^+^ T cells provide interclonal help to high-affinity CD8^+^ T cells to augment alloimmunity. Am J Transplant.

[32] Zareie P, La Gruta NL (2023). Thanks for the memories: low-avidity T cells shine against escape variants. Immunity.

[33] Zehn D (2009). Complete but curtailed T-cell response to very low-affinity antigen. Nature.

[35] Harris DT (2016). Deep mutational scans as a guide to engineering high affinity T cell receptor interactions with peptide-bound major histocompatibility complex. J Biol Chem.

[36] Luo F (2025). Functional avidity enhancement of a T-cell receptor targeting the KRAS^G12D^ cancer neoantigen. Cell Immunol.

[37] Murata K (2022). Modification of the HLA-A*24:02 peptide binding pocket enhances cognate peptide-binding capacity and antigen-specific T cell activation. J Immunol.

[38] Rosenberg AM (2024). Enhanced T cell receptor specificity through framework engineering. Front Immunol.

[39] Sun Y (2023). Universal open MHC-I molecules for rapid peptide loading and enhanced complex stability across HLA allotypes. Proc Natl Acad Sci U S A.

[40] Zhang M (2024). Identification and affinity enhancement of T-cell receptor targeting a KRAS^G12V^ cancer neoantigen. Commun Biol.

[41] Ruibal P (2022). Identification of HLA-E binding *Mycobacterium tuberculosis*-derived epitopes through improved prediction models. J Immunol.

[42] Nicolet BP, Wolkers MC (2022). The relationship of mRNA with protein expression in CD8^+^ T cells associates with gene class and gene characteristics. PLoS One.

[43] Shebl FM (2010). Comparison of mRNA and protein measures of cytokines following vaccination with HPV-16 L1 virus like particles. Cancer Epidemiol Biomarkers Prev.

[44] Weerakoon H (2024). Integrative temporal multi-omics reveals uncoupling of transcriptome and proteome during human T cell activation. NPJ Syst Biol Appl.

[45] Jouand N (2018). HCMV triggers frequent and persistent UL40-specific unconventional HLA-E-restricted CD8 T-cell responses with potential autologous and allogeneic peptide recognition. PLoS Pathog.

[46] Joosten SA (2016). Characteristics of HLA-E restricted T-cell responses and their role in infectious diseases. J Immunol Res.

[47] Prezzemolo T (2018). Detailed characterization of human Mycobacterium tuberculosis specific HLA-E restricted CD8^+^ T cells. Eur J Immunol.

[48] Tang K (2023). HLA-E-restricted Hantaan virus-specific CD8^+^ T cell responses enhance the control of infection in hemorrhagic fever with renal syndrome. Biosaf Health.

[49] Joosten SA (2010). Mycobacterium tuberculosis peptides presented by HLA-E molecules are targets for human CD8 T-cells with cytotoxic as well as regulatory activity. PLoS Pathog.

[50] Migueles SA (2023). HIV vaccines induce CD8^+^ T cells with low antigen receptor sensitivity. Science.

[51] van Meijgaarden KE (2015). Human CD8^+^ T-cells recognizing peptides from Mycobacterium tuberculosis (Mtb) presented by HLA-E have an unorthodox Th2-like, multifunctional, Mtb inhibitory phenotype and represent a novel human T-cell subset. PLoS Pathog.

[52] Cocchi F (1995). Identification of RANTES, MIP-1 alpha, and MIP-1 beta as the major HIV-suppressive factors produced by CD8+ T cells. Science.

[53] Temerozo JR (2023). Interleukin-27 promotes divergent effects on HIV-1 infection in peripheral blood mononuclear cells through BST-2/tetherin. J Virol.

[54] Boppana S (2019). HLA-I associated adaptation dampens CD8 T-cell responses in HIV Ad5-Vectored vaccine recipients. J Infect Dis.

[55] Qin K (2021). Elevated HIV infection of CD4 T cells in MRKAd5 vaccine recipients due to CD8 T cells targeting adapted epitopes. J Virol.

[56] Clutton GT (2020). CD3 and CD8 coreceptor down-modulation are inversely associated with CD8 T cell functional avidity in humans. J Immunol.

[57] Clutton GT (2021). CD8 co-receptor links T cell avidity and metabolism. J Immunol.

[58] Huang M (2019). FACS isolation of low percentage human antigen-specific CD8^+^ T cells based on activation-induced CD3 and CD8 downregulation. J Immunol Methods.

[59] Trimble LA (2000). Human immunodeficiency virus-specific circulating CD8 T lymphocytes have down-modulated CD3zeta and CD28, key signaling molecules for T-cell activation. J Virol.

[60] Trimble LA (2000). CD3zeta and CD28 down-modulation on CD8 T cells during viral infection. Blood.

[61] Files JK (2022). HLA-II-associated HIV-1 adaptation decreases CD4^+^ T-cell responses in HIV-1 vaccine recipients. J Virol.

[62] Currenti J (2021). Cross-reactivity to mutated viral immune targets can influence CD8^+^ T cell functionality: an alternative viral adaptation strategy. Front Immunol.

[63] Mayer-Blackwell K (2022). Flexible distance-based TCR analysis in Python with tcrdist3. Methods Mol Biol.

[64] Shannon P (2003). Cytoscape: a software environment for integrated models of biomolecular interaction networks Genome Res.

[65] Toebes M (2009). Generation of peptide MHC class I monomers and multimers through ligand exchange. Curr Protoc Immunol.

