# Supplementary material for: CD8+ T cells cross-restricted by HLA-B*57 and HLA-E*01 recognize HIV Gag with different functional profiles
Source: JCI Insight. 2025 Dec 18;11(3):e189909. doi: 10.1172/jci.insight.189909 (PMC12893105; doi:10.1172/jci.insight.189909)
Supplement: Supplemental data [file jciinsight-11-189909-s266.pdf]

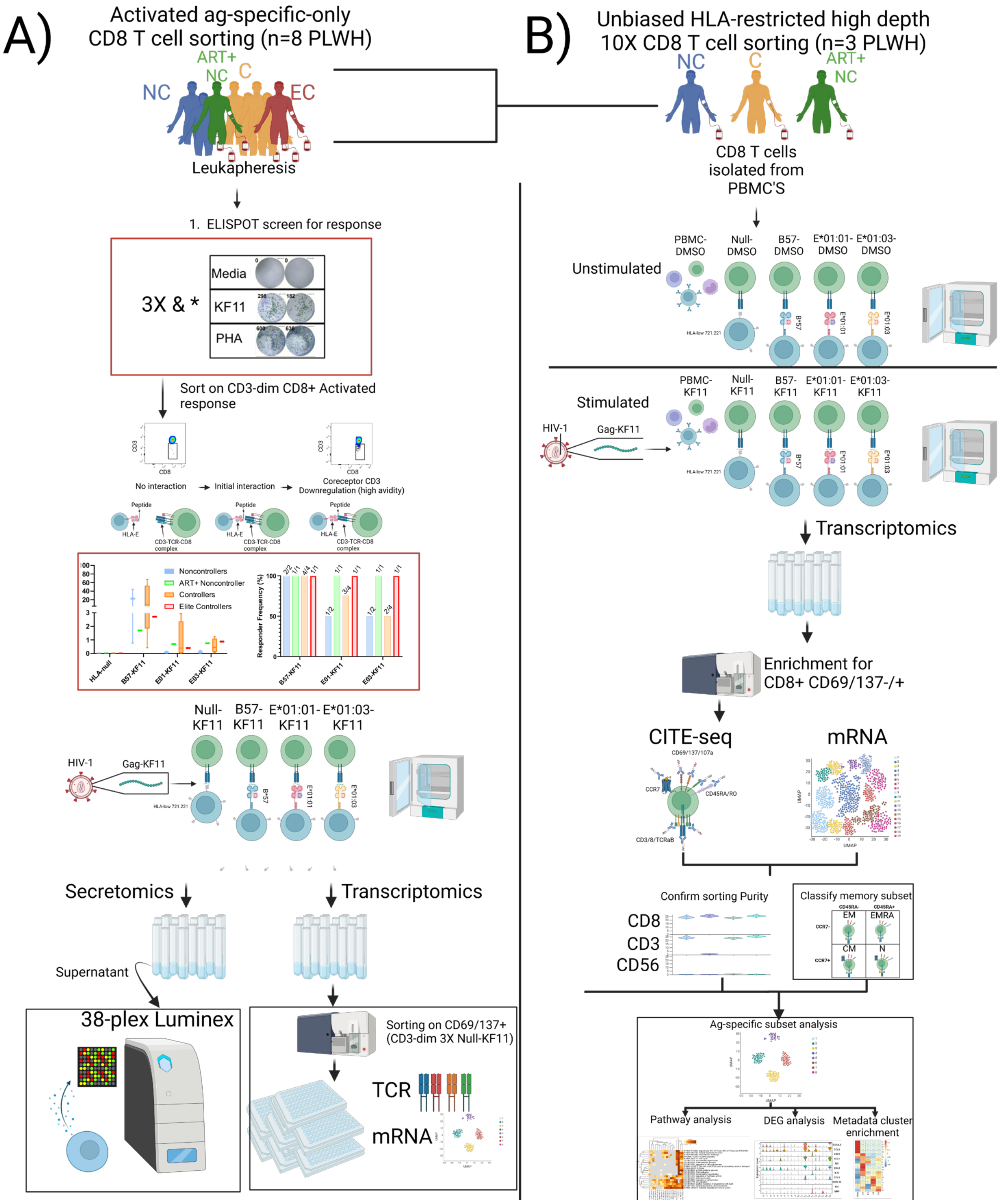

A)
Activated ag-specific-only
CD8 T cell sorting (n=8 PLWH)
NC
ART+ NC
C
EC
Leukapheresis
1. ELISPOT screen for response
3X & *
Media
KF11
PHA
Sort on CD3-dim CD8+ Activated response
No interaction
Initial interaction
Coreceptor CD3 Downregulation (high avidity)
Peptide
HLA-E
CD3-TCR-CD8 complex
Noncontrollers
ART+ Noncontroller
Controllers
Elite Controllers
Responder Frequency (%)
HLA-null
B57-KF11
E01-KF11
E03-KF11
Null-KF11
B57-KF11
E*01:01-KF11
E*01:03-KF11
HIV-1
Gag-KF11
Secretomics
Transcriptomics
Supernatant
38-plex Luminex
Sorting on CD69/137+ (CD3-dim 3X Null-KF11)
TCR
mRNA
B)
Unbiased HLA-restricted high depth
10X CD8 T cell sorting (n=3 PLWH)
NC
C
ART+ NC
CD8 T cells isolated from PBMC'S
Unstimulated
PBMC-DMSO
Null-DMSO
B57-DMSO
E*01:01-DMSO
E*01:03-DMSO
Stimulated
PBMC-KF11
Null-KF11
B57-KF11
E*01:01-KF11
E*01:03-KF11
HIV-1
Gag-KF11
Transcriptomics
Enrichment for CD8+ CD69/137-/+
CITE-seq
mRNA
CD69/137/107a
CCR7
CD45RA/RO
CD3/8/TCRaB
UMAP
Confirm sorting Purity
CD8
CD3
CD56
Classify memory subset
EM
EMRA
CM
N
Ag-specific subset analysis
Pathway analysis
DEG analysis
Metadata cluster enrichment

Experimental and analysis workflow graphical abstract: Graphical abstract generated through Biorender illustrating the 2 sets of experiments used to obtain the data in this study. A set of 8 PLW leukopheresis donor samples (2 NC, 1 ART+ NC, 4 C, and 1 EC) were used for all experiments. (Left side) Initial targeted antigen-specific scRNA/TCR-seq sorting and 38-plex Luminex strategy. (Right side) Follow-up higher-depth and broader/unbiased condition sorting strategy for 10X. Both unstimulated and stimulated (KF11 unpulsed or pulsed) conditions for all previous HLA's (null, B57, E01, E03) were utilized alongside PBMC's. Both enriched activated and unactivated CD8s were sorted to include resting memory repertoire and achieve sufficient cell number. These cells were multiplexed by patient and subjected to 10X multi-omic sequencing for scTCR, scRNA, and scCITE-seq expression data acquisition. The TCR data was analyzed through a novel Chi-squared approach to determine which TCR α/β paired families and clonotypes significantly increased in every combination of metadata from unstimulated to stimulated, and these significantly increasing pairs were then interrogated through metaclonotype clustering by dissimilarity distance using TCRDist3, clusters which were then compared to those obtained in the targeted ag-specific analysis through the previous strategy. First, cells were assayed for cell marker surface expression through CITE-seq to ensure purity of CD56- CD8+ T cell sorting. Cells were then subset by those whose TCR pairs matched the ones found to significantly increase in some condition/combination of conditions and classified into memory subsets based on their surface expression of CD45RA and internal expression of CCR7 and these clusters were interrogated for differential gene expression, gene ontology, and metadata-driven analysis.

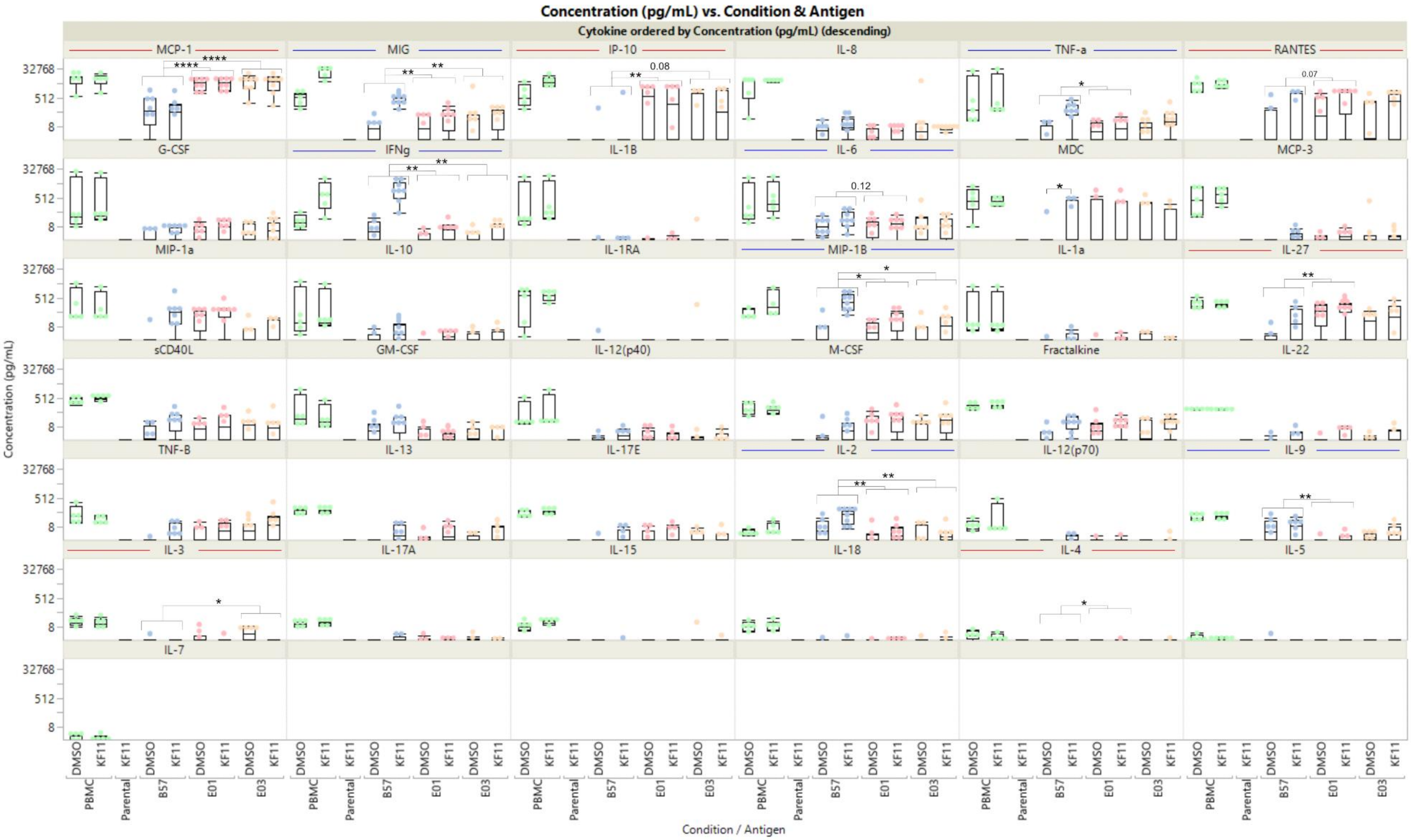

Supplemental Figure 2: Muliplex-ELISA Cytokine and chemokine Response Summary. Summary of all 8 HLA-class I restricted responses to either no peptide or KF11 across all responders, cytokines separated by boxes. All concentrations for each donor in each condition are normalized to Parental-cell line KF11 response (41A3.CD4 "null" cell line pulsed with KF11 and co-cultured with CD8s from all 8 donors) individually except for PBMC response. Higher HLA-B57-restricted or -E*01:01 response is represented by a blue or red line respectively.

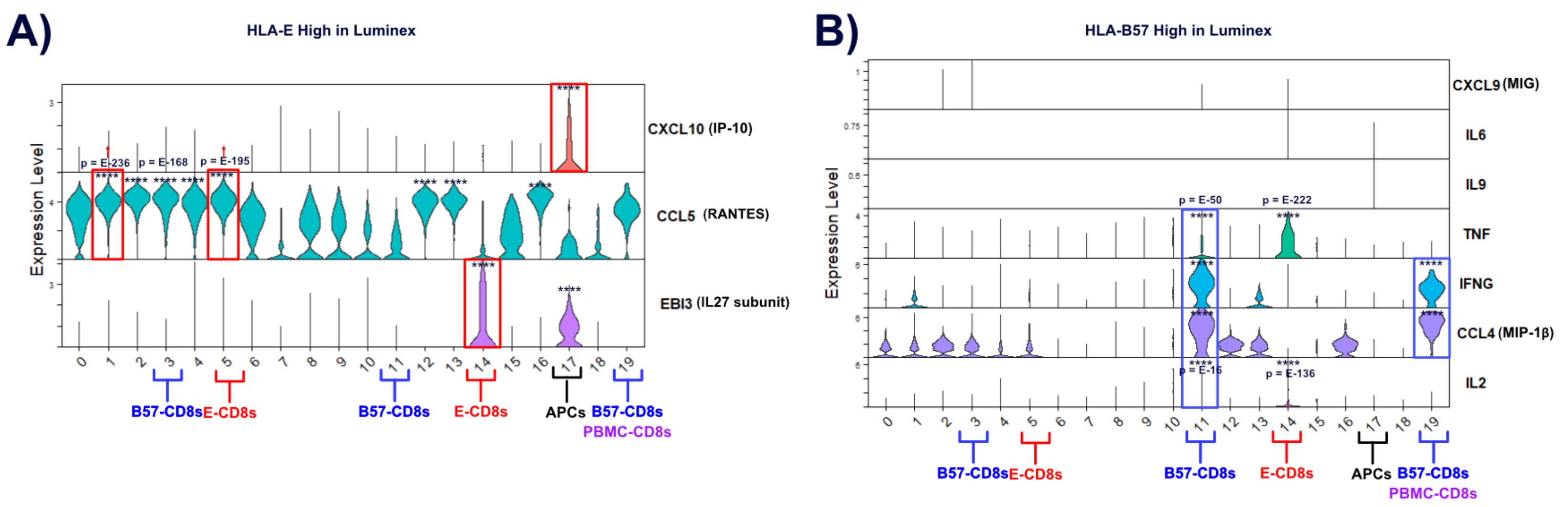

Supplemental Figure 3: Several secreted Cytokines and chemokines Confirmed to be Expressed within Appropriate HLA-restricted CD8s. Violin plot of RNA expression for those cytokines identified through high-parameter Luminex in HLA-restricted co-culture within 10X Seurat clusters. **A)** HLA-E- or **B)** -B57-restricted cytokines which are significantly increasing compared to all other clusters by MAST are boxed in red or blue respectively. Relevant clusters are marked as E-CD8s or B57-CD8s below depending on relative number of cells for HLA-restricted metadata in those clusters. Exact p values are given when given cytokine is positive in more than 1 cluster and in both an —CD8 and B57-CD8 cluster for comparison. All p values given are FDR-corrected (p = 0.05, 0.01, 0.001, 0.0001 represented as *, **, ***, **** respectively) .

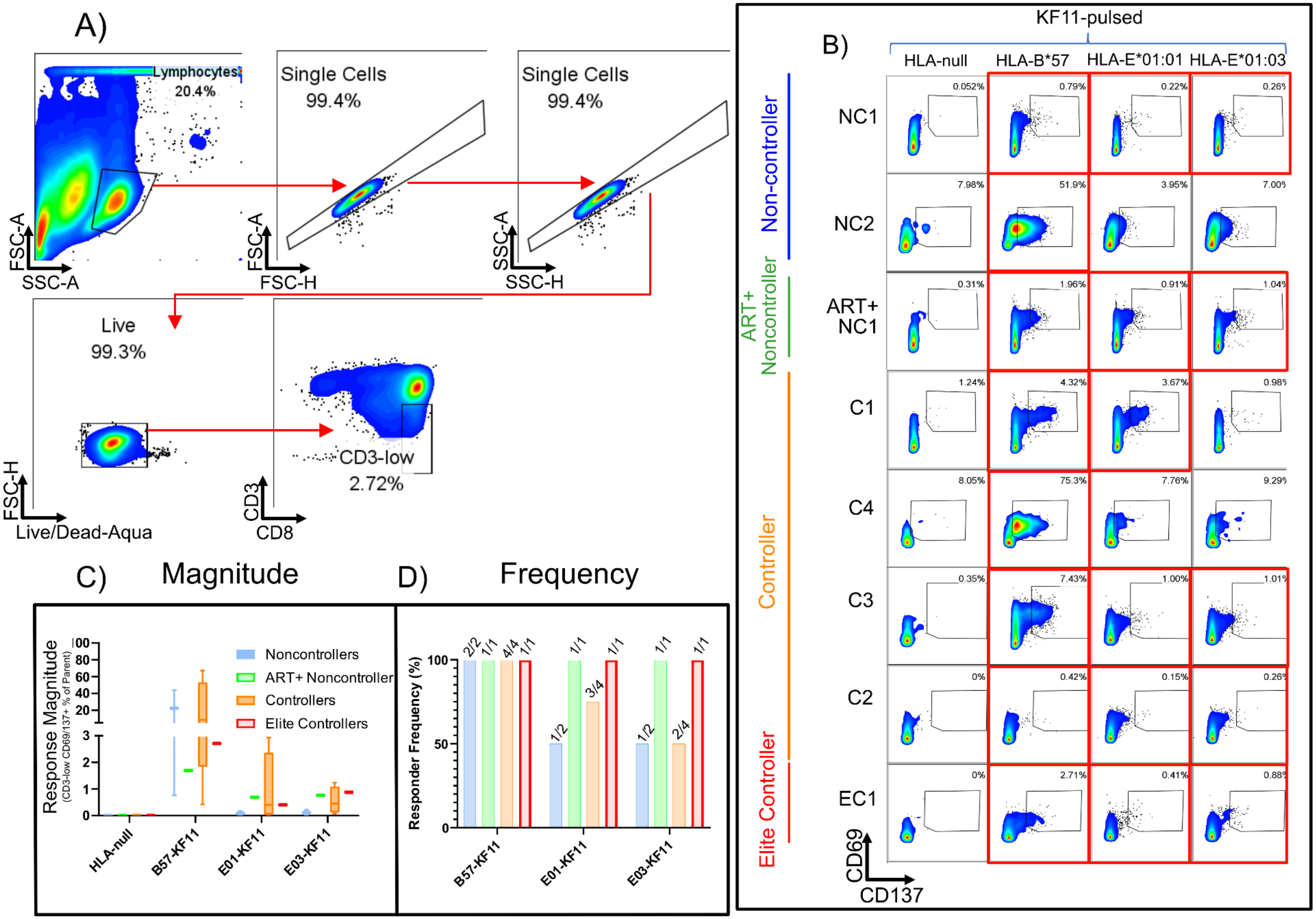

Supplemental Figure 4: Gating strategy and response magnitude for CD3-low HLA-class I restricted responses. A) Gating strategy for identifying CD3-low CD8+ T cells. **B)** Response magnitude of dually activated (CD69+ CD137+) CD3-low high avidity HLA-restricted CD8+ T cells to KF11 in each HLA group. **C)** Graph showing magnitude of CD69+ CD137+ response shown in (B). **D)** Bar graph showing response frequency among noncontrollers, ART-suppressed noncontroller, controllers, and elite controller in cohort relative to HLA-B*57:01-, E*01:01-, and E*01:03-restricted KF11 from (B).

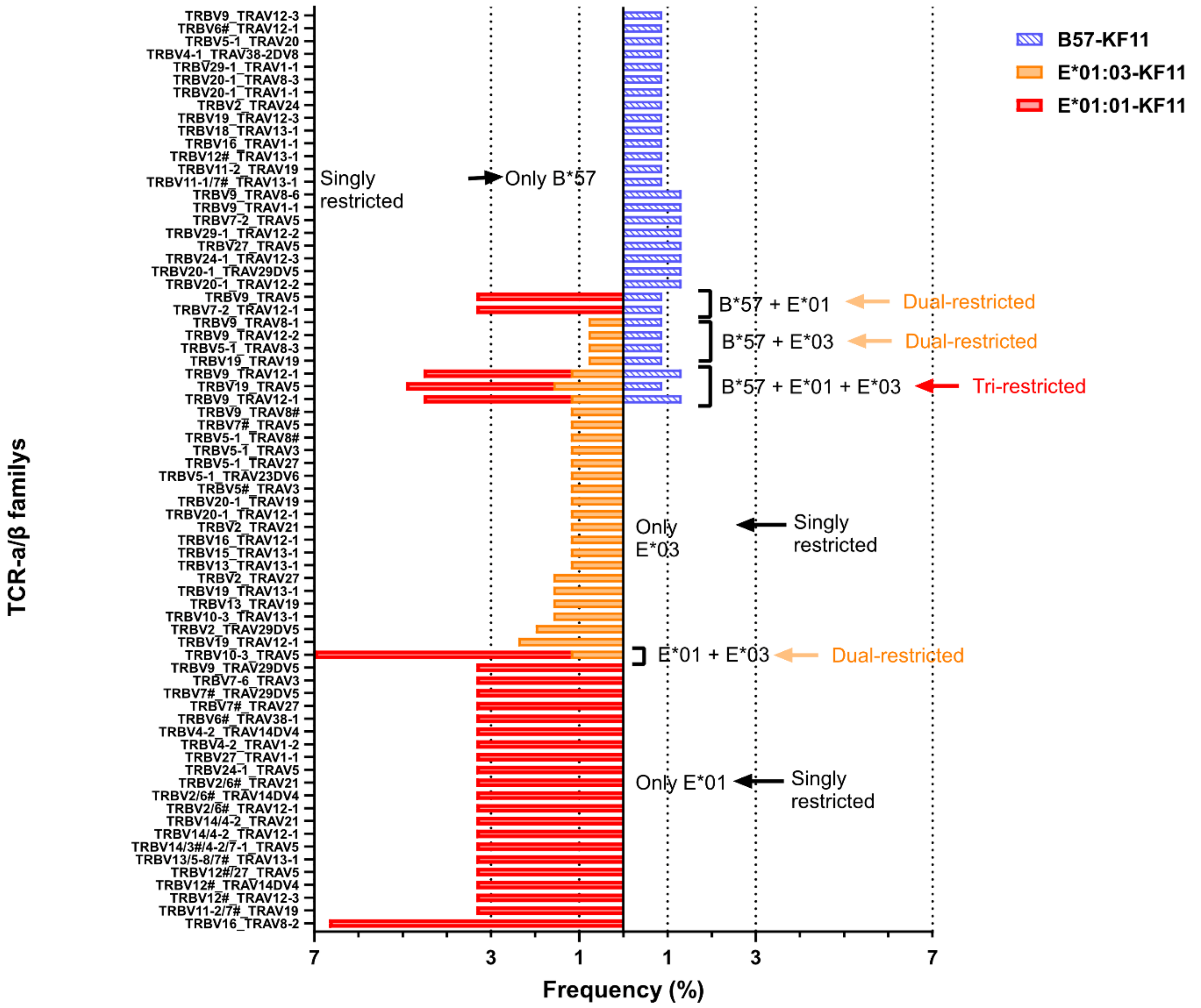

Supplemental Figure 5: Pyramid plot of TCR cross-restriction in one PWH. Pyramid plot in same style as Figure 2A, but for a single PWH to show cross-restriction at the donor level in activated CD8s.

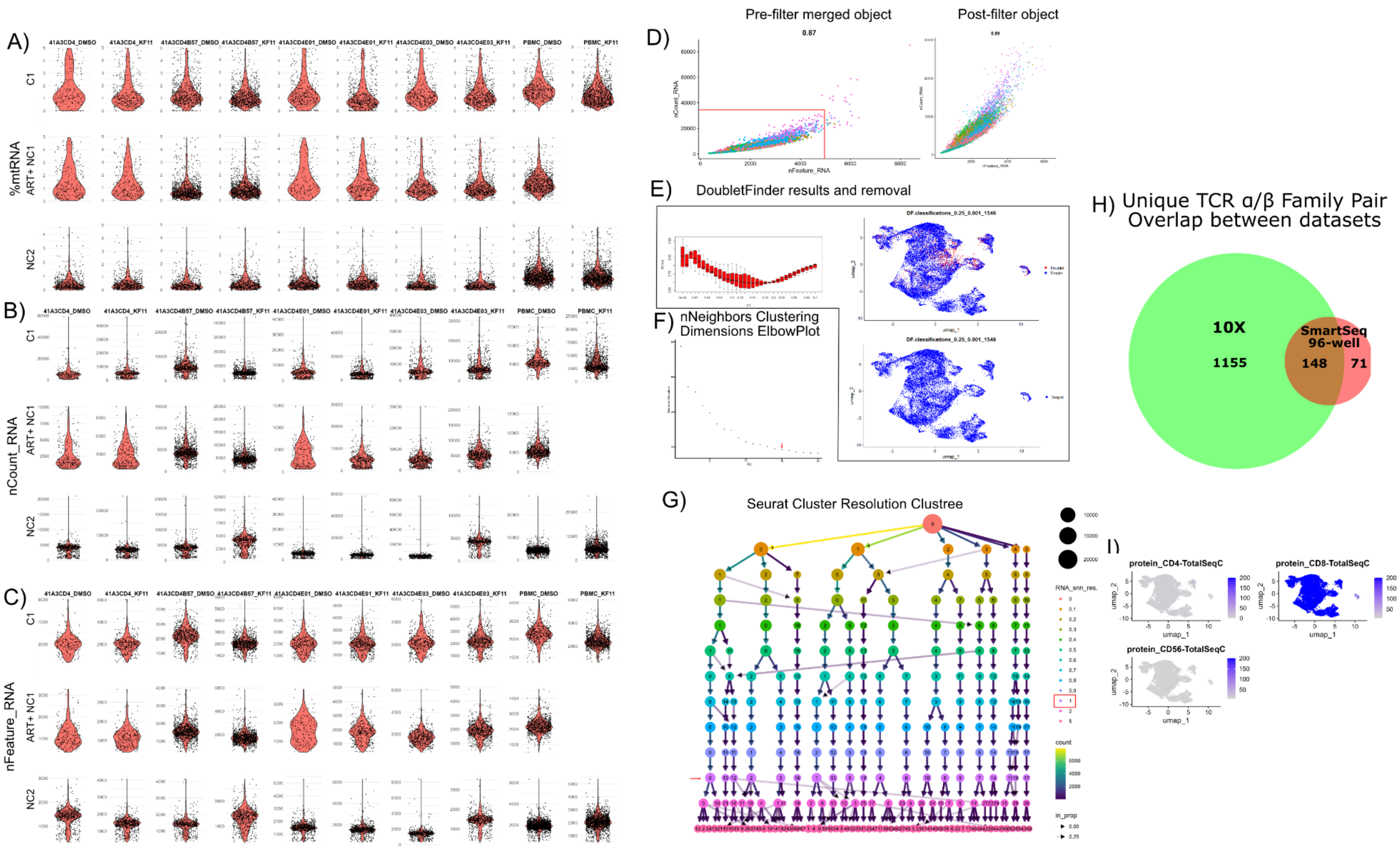

Supplemental Figure 6: 10X genomics QC metrics. Violin plots by donor (rows) and condition (columns) for post-filtered **A)** %miRNA, **B)** nCount_RNA, and **C)** nFeature_RNA values of merged dataset are shown. **D)** Scatterplot showing nCount_RNA to nFeature_RNA pre- and post-98th percentile filtering in an attempt to remove outliers from dataset. Additional **E)** DoubletFinder filtering used to more specifically identify and remove potential doublets. Left boxplot of BCReal vs pK simulation used to find optimal pK setting in package to identify potential doublets within dimplot shown at upper right. Lower-right dimplot represents post-filtered merged dataset. **F)** Elbowplot was used to determine optimal number of dimensions on which to plot the UMAP. **G)** Clustree used to determine optimal resolution which best identified distinct clusters of transcriptionally distinct CD8s that were not “overclustered”, or do not have substantial cross-assignment to non-existent distinct clusters, as represented by non-opaque arrows. **H)** Overlap of unique TCR α/β family pairs observed in antigen-specific CD3-low high avidity scTCR-seq data and 10X scTCR-seq data.

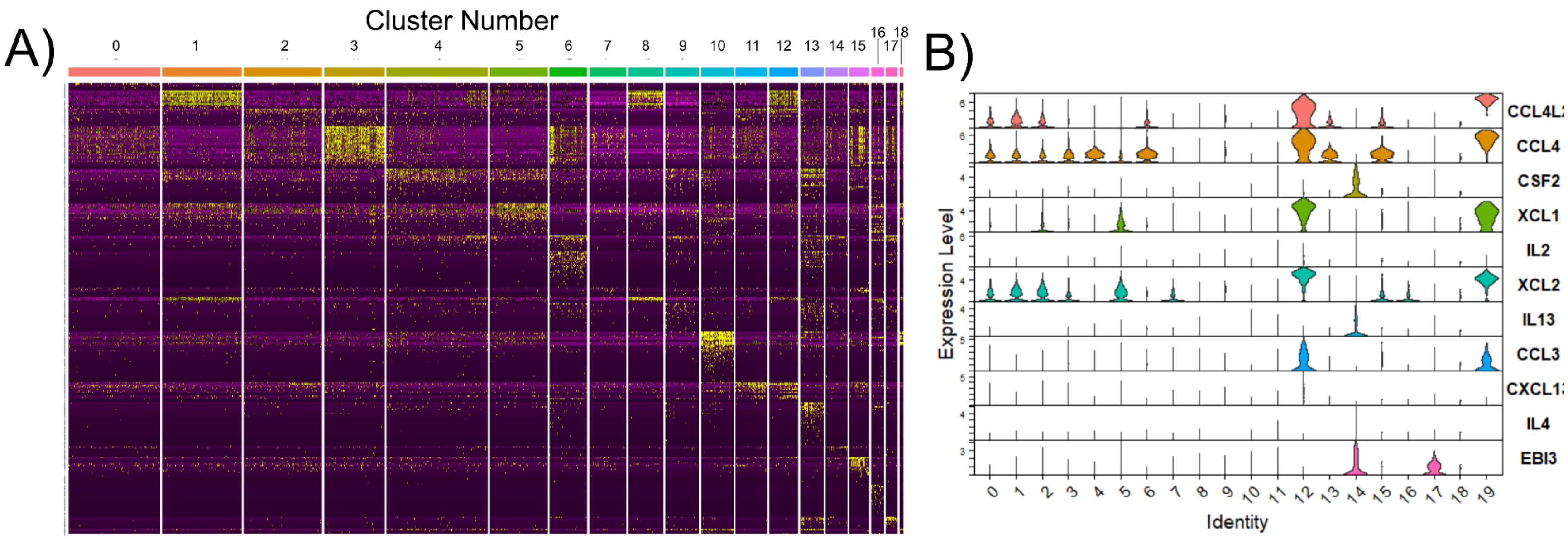

Supplemental Figure 7: Clusters have distinct transcriptional profiles. A) Dim Heatmap of top 50 markers identified per cluster by MAST, unlabeled. **B)** Violinplot of top variable cytokines across dataset separated by cluster.

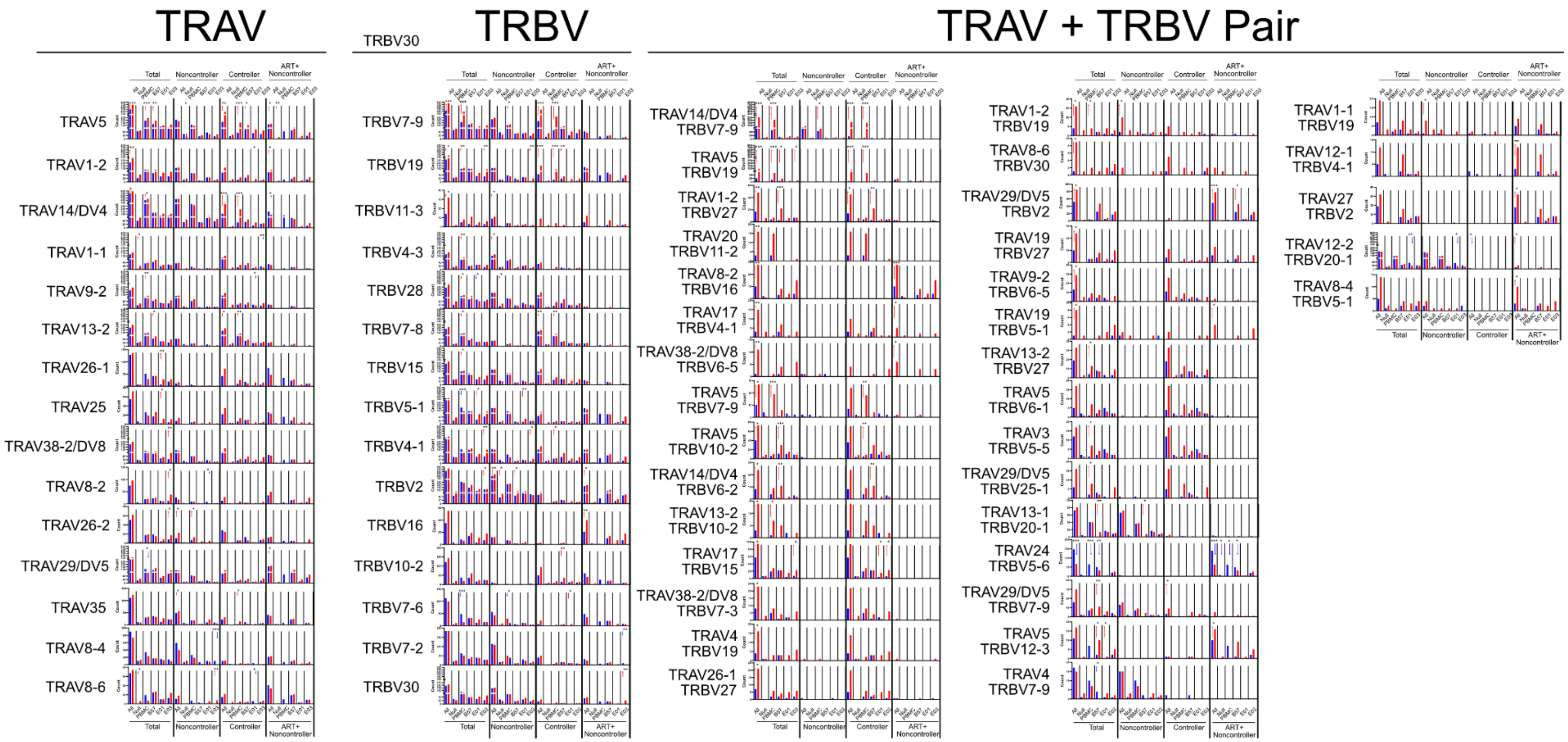

Supplemental Figure 8: Stacked bar graph summary of significantly increasing α, β, or α/β paired families. Bar graphs of significantly increasing A) α, B) β, or C) α/β paired families showing count of each family or paired family as blue for unstimulated or red for stimulated, separated by first control status (Total, noncontroller, controller, ART-suppressed noncontroller) and sub-set by HLA status within those control statuses (All, Null, PBMC, B57, E01, and E03). Red up arrow and blue down arrow indicates increasing or decreasing family within the indicated metadata combination. Significance determined through novel Chi-squared comparison of count of cells between unstimulated and stimulated compared to counts of indicated family or family pair between unstimulated and stimulated metadata combination. ART-supprssed noncontroller PBMC data not available due to sorting error (1 out of 30 conditions), and so no significance applicable in that condition. Significance of increase or decrease indicated above (*, **, ***, **** mean $p < 0.05$, 0.01, 0.001, 0.0001 respectively).

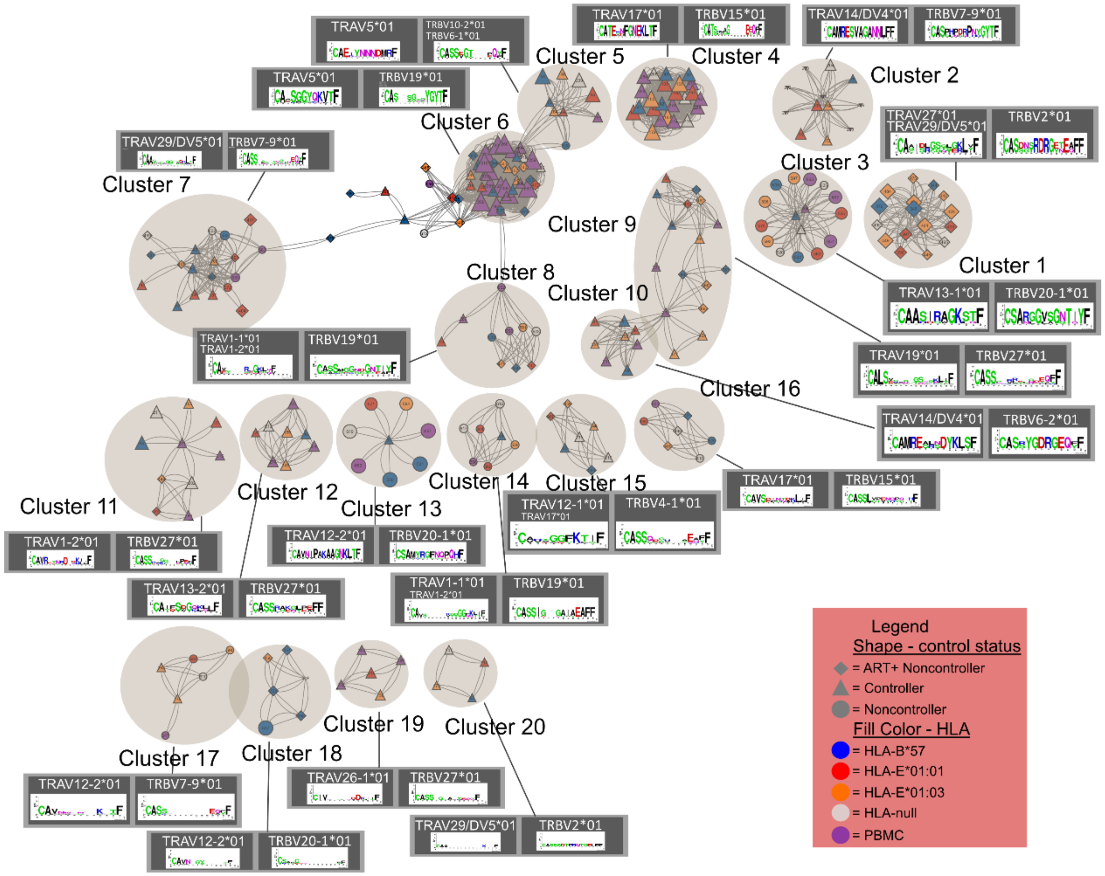

Supplemental Figure 9: Antigen-specific clonotypes are multiply HLA-I restricted. TCRdist3 distance matrix-based clustering of KF11 stimulated significantly increasing α/β pair family clonotypes above proportion and TDU cutoffs. With increased cell number and therefore cluster number, clusters are labeled for reference in text. TCR families included in each cluster are also labeled by cluster alongside a Logo plot detailing the CDR3 sequences therein. PBMC, Null-, HLA-B*57:01-, E*01:01-, and E*01:03-restricted clonotypes are colored in purple, gray, blue, red, and orange respectively. Clonotypes identified in noncontrollers are represented as circles, while those in controllers and the ART-suppressed noncontroller are represented as triangles and diamonds respectively.

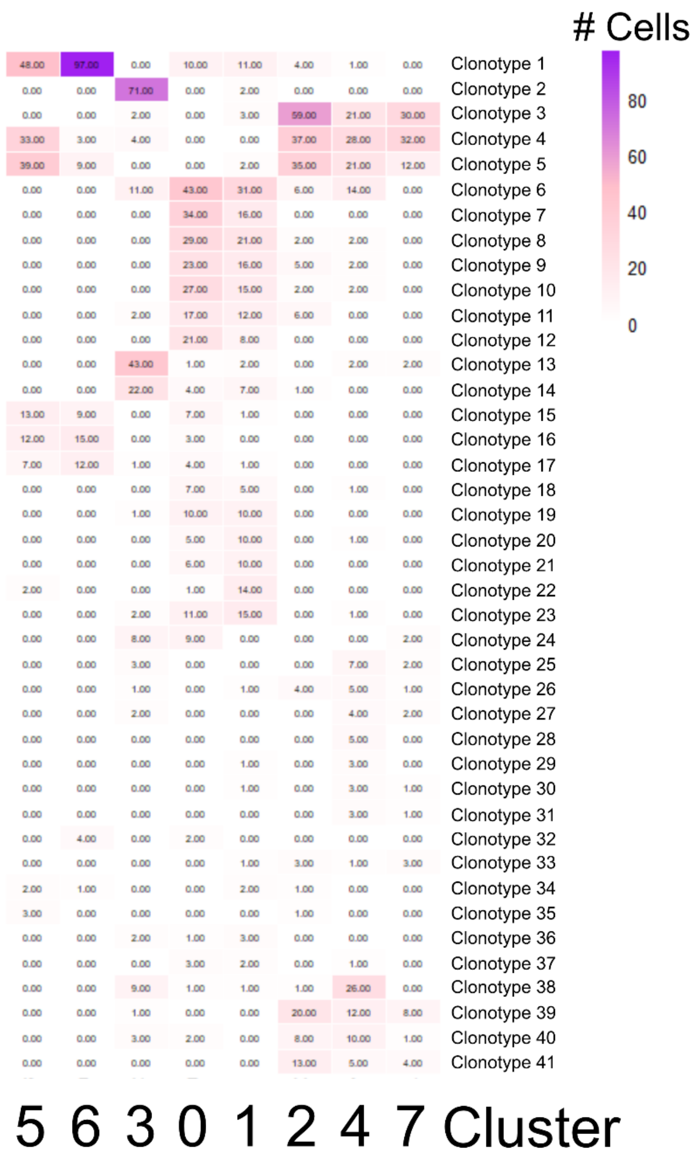

Supplemental Figure 10: Antigen-specific HLA-restricted clonotypes are transcriptionally distinct from one another. Heatmap of number cells with paired TCR clonotype sequence metadata (unique clonotype paired sequence 1, 2, 3, etc.) found within each Seurat cluster within the ag-specific subset of 10X data.

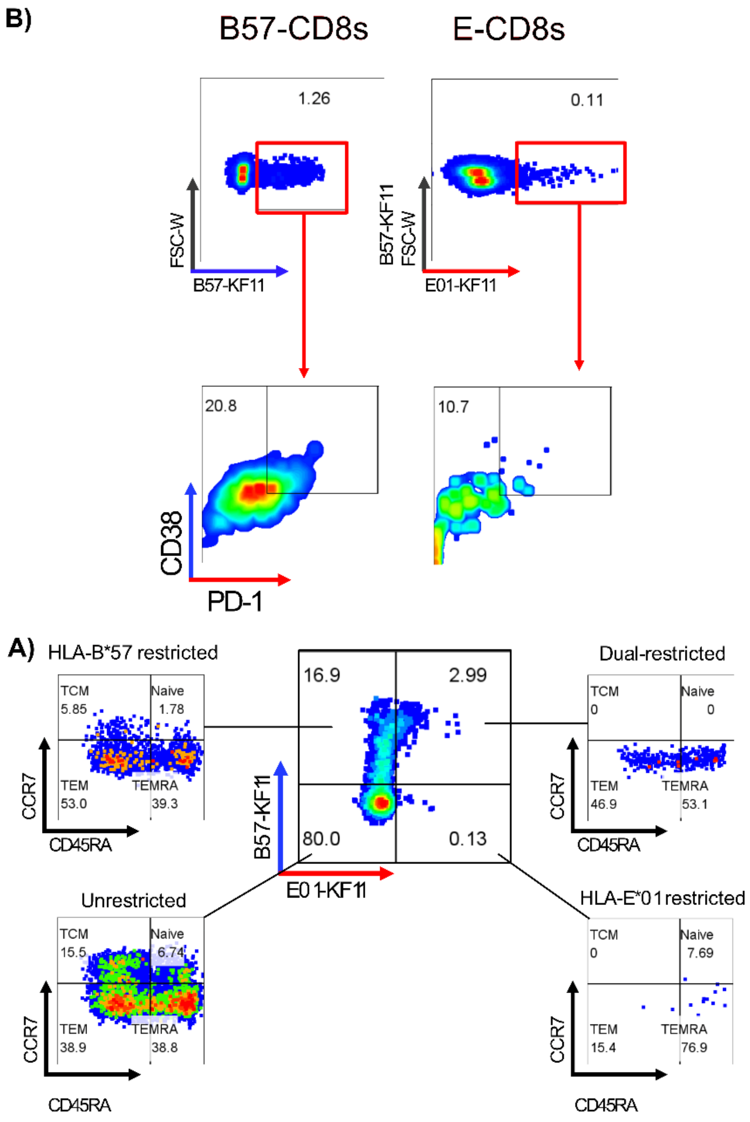

Figure S11: Gating for Dextramer staining based examination of CD8 Memory and Exhaustion. A) Representative gating for CD38+ PD1+ exhaustion within all B57-CD8s or E-CD8s within cohort. **B)** Memory state of all 4 quadrants of B57+/- and E01-KF11 +/- dextramer PWH EC2 αβ. Representative graph of memory state (CD45RA vs CCR7) within each dextramer-positive quadrant shown. Same gating used to determine memory state across rest of cohort.

Supplemental Table 1: Cohort demographics. Table for all donor samples used in study. Control status determined as stated in text for noncontrollers (NC), controllers (C), and elite controllers (EC) having a plasma viral load >2000 , <2000, or <200 cp/mL respectively. "KF11 ELISPOT IFNγ SFU/1e6 cells" shows ELISPOT IFNγ response magnitude of indicated donor PBMCs to KF11 peptide if tested based on sample availability.

Status	Alternate ID	Viral Load (cp/mL)	HLA-A1	HLA-A2	HLA-B1	HLA-B2	KF11 ELISPOT IFNγ SFU/1e6 cells
C 1	Ci10004	253	A*03:01	A*30:02	B*07:02	B*57	2400
C 2	Ci10060	722	A*01:01	A*33:01	B*42:01	B*57	415
C 3	Ci10067	215	A*30:02	A*33:01	B*13:02	B*57	610
C 4	Ci20018	1886	A*02:01	A*26:01	B*40:01	B*57	1520
EC 1	Ci10071	49	A*01:01	A*66:02	B*8:01	B*57	730
ART+ NC	Ci10074	49	A*01:01	A*26:01	B*8:01	B*57	770
NC 1	Ci10027	4827	A*01:01	A*02:01	B*8:01	B*57	714
NC 2	Ci10076	17693	A*02:01	A*30:02	B*35:01	B*57	N/A
EC 2	8219	47	A*02:01	A*30:02	B*07:05	B*57	2737
PWO 1		0	A*30:01	A*68:02	B*42:01	B*57	N/A

Supplemental Table 2: Antibody List - Sorting. Table for all antibodies describing the marker, fluorophore, clone, company source, and catalog number for the antibody “sorting mix” referenced in Methods section.

Marker	Fluorophore	Clone	Company	Catalog #
Live-dead	Aqua	N/A	Invitrogen	L34957
CD3	PacBlue	UCHT1	BD	558117
CD4	A780	RPA-T4	Invitrogen	47-0049-42
CD8	A700	RPA-T8	BD	561453
CD14	PerCP-Cy5.5	M5E2	BD	550787
CD16	PerCP-Cy5.5	3G8	BD	560717
CD19	PerCP-Cy5.5	SJ25C1	BD	340951
CD56	PerCP-Cy5.5	B159	BD	560842
CD69	APC	FN50	BD	555533
CD137	PE	4B4-1	BD	555956
CD94	PE-Cy7	DX22	Biolegend	305516
CD107a	FITC	H4A3	BD	555800

Supplemental Table 3: Antibody List – Multimer Testing. Table for all antibodies describing the marker, fluorophore, clone, company source, and catalog number for the antibody “multimer staining mix” referenced in Methods section.

Marker	Fluorophore	Clone	Company	Catalog #
Live-dead	Zombie Aqua	N/A	Invitrogen	L34957
CCR7	RealBlue 705	3D12	BD	756922
CD3	A780	UCHT1	Invitrogen	47-0038-42
CD4	BV711	SK3	Biolegend	344648
CD8	PE-Texas Red	3B5	Invitrogen	MHCD0817
CD14	BUV563	MϕP9	BD	741441
CD16	BUV563	B73.1	BD	741449
CD19	BUV563	SJ25C1	BD	612916
CD56	BUV805	B159	BD	742022
CD94	PE-Cy7	DX22	Biolegend	305516
CD38	StarBright Blue 615	AT13/5	BioRad	MCA1019SBB615
PD1	BV650	J105	Invitrogen	41-627-9942
CD45RA	StarBright Blue 765	F8-11-13	BioRad	MCA88SBB765
KLRC1/NKG2A	mFluor Violet 610	131411	Novus	FAB1059MFV610
KLRC2/NKG2C	A700	134522	R&D Systems	FAB1381N
TCR-a/B	BUV737	IP26	BD	749196
CD57	BV785	QA17A04	Biolegend	393330

Supplemental Methods

10X Analysis pipeline

10X Demultiplexing. Initially, 3 sets of paired reads fasta files were obtained for the 3 types of data (RNA EXPRESSION, TCR, and CITE-seq/multiplex hashtag antibody feature barcodes (FB)) for each multiplexed donor for a total of 18 files and 112 GB of gzipped data. Initial de-multiplexing was performed on these 3 donor multiplexed data using the RNA EXPRESSION and hashtags of the FB fasta files adopting a modified version of the strategy described here so as to in the most unbiased manner use the 5′ Biolegend hashtagging oligos with the 3′ CMO Biolegend demultiplexing pipeline to assign cells to different samples within each donor:https://www.10xgenomics.com/resources/analysis-guides/demultiplexing-and-analyzing-5%E2%80%99-immune-profiling-libraries-pooled-with-hashtags. A hashing_demux_HMO-set.csv targets file as well as a demultiplex config file were used for this purpose. After initial QC of the number of cells and reads returned for every sample, it became apparent that the elite controller PBMC-KF11 stimulated condition was either not sorted or not amplified. Bam files generated in the demultiplexing process were then converted back to fastq within donor sample folders under condition folders and TCR and CITE-seq data was then extracted using the cell id's assigned to each sample from demultiplexing using cellranger multi and a new config file for each sample within each donor directory. This resulted in 87 unique fasta files, 3 each RNA EXPRESSION, TCR, and FB for the 10 conditions per donor sample under donor/condition directory folder, then used for downstream Seurat analysis.

Initial pre-processessing. In brief, a loop was used based on the filestructure naming scheme and both patient as well as condition names to individually construct Seurat objects using CreateSeuratObject, then assigning metadata to each individual object based on the condition and patient names of the filestructure. Initially, RNA EXPRESSION was first imported under the "RNA" assay object, after which FB (containing the CITE-seq expression data) was imported into the "Protein" assay object. Each 10 (or 9 for the ART$^+$ NC where PBMC-KF11 data was not present) condition objects were then added to SeuratLists by donor within the loop. Metadata was then assigned using another loop based on the naming scheme of these Seurat objects inside their SeuratLists for HLA, donor, control status, and other relevant metadata.

TCR metadata pre-processing (VCR-Seek). TCR data was processed similarly, but instead of being used to create Seurat objects, individual "filtered_contig_annotations.csv" files output from cellranger under each folder in the filestructure were individually imported. MixCR format was, as always, incompatible with all downstream analysis due to data being separated into rows based on TCR α or β chain rather than by cell, so based on the barcode ID, these were combined into a single row with new column names by cell. This data was then analyzed individually within that sample for proportions of each chain or paired chain as well as CDR3 chain or paired clonotype before then being attached as new metadata columns to the relevant Seurat object within its SeuratList. Unique prefixes were then applied for each individual cell by patient_condition combination. TCR meta-analysis was performed by extracting this metadata (both TCR and other metadata such as stimulation status, hla condition, and control status from each object and combining all of it into a single data table as "combined_metadata.csv." Proportions of each TCR αβ individual or paired chain CDR3 clonotype or family for every combination of metadata (control, HLA, stimulation, all or sub-combination of any of these) was calculated across all cells matching that metadata combination. Counts of each of those single or paired chain family or CDR3 sequences were then also tabulated by all combinations. Delta proportions for each combination from DMSO to KF11 stimulation conditions were then calculated and a Chi-squared test was performed for every single or paired chain between stimulation conditions to determine whether a significant delta increase or decrease was observed. These results were condensed to only unique chain families or CDR3 sequences (single or paired) as separate tabs of an output excel spreadsheet, separated by α, β, or paired αβ. A new sheet containing curated significantly increasing TCR's from this output was reformatted for TCRdist3 analysis as previously. Family and clonotype analysis were performed in separate steps. Clonotype id's as determined across all data (not within each condition) were then assigned to the downstream merged SeuratObject for all samples.

RNA expression pre-processing and QC. Initial pre-processing of gene-expression analysis was carried out by first iterating through each Seurat object within each list and Log normalizing and scaling the data using the RNA expression data before finding variable features and filtering out any cells which had mtRNA% < 5 and nFeature_RNA > 200 (Suppelemental Figure 4, A–C). QC metrics plots for these as well as nCountRNA were generated after this step to visualize distribution of data within samples and ensure cutoffs were successfully executed with the cowplot package. Because each individual sample was its own either control status or HLA condition and there was virtually no batch effect (all library creation and sequencing was performed immediately post sorting and within 1 week of eachother), integration was not pursued as a method to generate a single unified Seurat object containing all data across all samples. Within each patient SeuratList, all condition Seurat objects were merged into a single patient Seurat object (3 objects, 1 per patient). These objects were then merged to form a single merged Seurat object for all samples containing all metadata, to which clonotype id information was applied to each cell based on the cdr3_a_b_aa pair sequence. nFeature and nCount upper limit cutoffs were at this point applied as QC for doublets, with the cells possessing top 2% quantile of each filtered out as outliers (Suppelemental Figure 4D). As an additional quality control, DoubletFinder was used to detect doublets based on simulated data comparisons, with the optimal pK of 0.001 and pN of 0.25 used as determined by highest average BCReal value (Suppelemental Figure 4E). To determine the number of dimensions on which to run nNeighbors and generate UMAPs, an ElbowPlot was used, after which the first 15 dimensions were found to be optimal (Suppelemental Figure 4F). The Clustree package was then used to determine the optimal resolution to generate Seurat clusters such that indeterminant cross-transfer was minimized (to prevent over-clustering) (65).

CITE-seq expression pre-processing into metadata

Utilizing the CITE-seq markers for CD45RA and the mRNA expression level of CCR7 (CITE-seq staining of CCR7 was suboptimal and did not match mRNA expression) across Seurat clusters, clusters were subset into naïve (N), effector memory (EM), terminal effector memory re-expressing RA (TEMRA), and central memory (CM) with the same "gating strategy" based on expression level above or below a percentile cutoff based on the same gating strategy by which these states would be determined in conventional flow cytometry (CCR7_, CD45RA_ +/+, –/–, +/–, –/+ denoting N, EM, CM, or TEMRA respectively). A similar methodology was used for determination of activation status, wherein triple positivity of CITE-seq CD69, CD137, and CD107a expression above an expression threshold cutoff was used to denote "activated" or "unactivated" status of a given cell. Metadata for HLA restriction, control status, stimulation status, and derived activation and memory state status was used to denote cluster metadata status based on relative. In brief, the number of cells corresponding to each state was output into a table format and converted into Z-scores within a heatmap scaled by cluster (as opposed to scaled to all clusters). This Z-score was then used to determine the metadata status of this cluster. For example, if Z-scores for number of B57- and PBMC- metadata CD8s were within 0.6 of one another but higher than 0.6 over all other HLA statuses for a given cluster, this cluster would be denoted B57_PBMC, with the same process used for all other metadata types in each cluster. This ensured the absence of bias, similar to the expression level cutoff.
